# Cancer Reversion Therapy: Prospects, Progress and Future Directions

**DOI:** 10.3390/cimb47121049

**Published:** 2025-12-15

**Authors:** Emmanuel O. Oisakede, David B. Olawade, Oluwakemi Jumoke Bello, Claret Chinenyenwa Analikwu, Eghosasere Egbon, Oluwaseun Fapohunda, Stergios Boussios

**Affiliations:** 1 Department of Clinical Oncology, Leeds Teaching Hospitals Trust, Leeds LS9 7TF, UK; emmanuel.oisakede@gmail.com; 2 Department of Health Research, University of Leeds, Leeds LS2 9JT, UK; 3 Department of Public Health, York St John University, London E14 2BA, UK; 4 Department of Business, Management and Health, School of Health, Sport and Bioscience, University of East London, London E16 2RD, UK; 5 Department of Research and Innovation, Medway NHS Foundation Trust, Gillingham ME7 5NY, UK; 6 The Clinical Research Centre, the London Clinic, 20 Devonshire Place, London W1G 6BW, UK; k.bello01@outlook.com; 7 Department of Microbiology, University Hospital Southampton NHS Foundation Trust, Hampshire SO16 6YD, UK; chinenyecollette@yahoo.com; 8 Department of Tissue Engineering and Regenerative Medicine, Faculty of Life Science Engineering, FH Technikum, 1200 Vienna, Austria; eghosaseregabriel@gmail.com; 9 Department of Chemistry and Biochemistry, University of Arizona, Tucson, AZ 85721-0041, USA; ofapohunda@arizona.edu; 10 Faculty of Medicine, School of Health Sciences, University of Ioannina, 45110 Ioannina, Greece; 11 Department of Medical Oncology, Ioannina University Hospital, 45500 Ioannina, Greece; 12 Faculty of Medicine, Health and Social Care, Canterbury Christ Church University, Canterbury CT1 1QU, UK; 13 Faculty of Life Sciences & Medicine, School of Cancer & Pharmaceutical Sciences, King’s College London, Strand, London WC2R 2LS, UK; 14 AELIA Organization, 9th Km Thessaloniki—Thermi, 57001 Thessaloniki, Greece

**Keywords:** cancer reversion, cellular reprogramming, differentiation therapy, tumor microenvironment, epigenetic regulation

## Abstract

Cancer reversion therapy represents a paradigm shift in oncology, focusing on reprogramming malignant cells to a non-malignant state rather than destroying them. This narrative review synthesizes current evidence, emerging technologies, and future directions in this promising field. Cancer reversion is founded on key biological observations: somatic cell reprogramming, spontaneous cancer regression, and microenvironmental influences on malignant behavior. Current approaches include epigenetic reprogramming using HDAC inhibitors and DNA methyltransferase inhibitors; microenvironmental modulation through extracellular matrix manipulation and vascular normalization; differentiation therapy exemplified by all-trans retinoic acid in acute promyelocytic leukemia; and targeting oncogene addiction as demonstrated in BCR-ABL-driven leukemias. Emerging technologies accelerating progress include single-cell analyses that reveal cancer heterogeneity and cellular state transitions; CRISPR-based approaches enabling precise genetic and epigenetic manipulation; patient-derived organoids that model tumor complexity; and artificial intelligence applications that identify novel reversion-inducing agents. Critical evaluation reveals that many reported “reversion” phenomena represent stimulus-dependent plasticity or transient growth arrest rather than stable phenotypic normalization. True cancer reversion requires durable, heritable phenotypic changes that persist after treatment withdrawal, with evidence of epigenetic consolidation and functional restoration. Despite promising advances, significant challenges remain: cancer cell plasticity facilitating therapeutic escape, difficulties in establishing stable reversion states, delivery challenges for solid tumors, and the need for combination approaches to address tumor heterogeneity. Future directions include integrated multi-omics analyses to comprehensively map cellular state transitions, studies of natural regression phenomena to identify reversion mechanisms, advanced nanodelivery systems for targeted therapy, and synthetic biology approaches creating intelligent therapeutic systems. By redirecting rather than destroying cancer cells, reversion therapy offers the potential for reduced toxicity and resistance, potentially transforming cancer from a deadly disease to a manageable condition.

## 1. Introduction

Cancer remains one of the most formidable challenges in modern medicine, with conventional treatments often leading to significant side effects, treatment resistance, and recurrence [1,2,3]. Despite remarkable advances in surgery, chemotherapy, radiotherapy, immunotherapy, and targeted therapies, we continue to search for more effective and less harmful approaches to cancer treatment. In this context, a paradigm-shifting concept has emerged in recent years: cancer reversion therapy.

Unlike traditional approaches that focus primarily on destroying cancer cells, cancer reversion therapy aims to reprogram malignant cells back to a normal or near-normal state [4]. This innovative strategy is built upon the growing understanding that cancer is not simply a disease of uncontrolled growth but rather a complex cellular state that may, under certain conditions, be reversible [5]. The concept challenges the long-held belief that genetic mutations in cancer are irreversible and suggests that phenotypic normalization might be achievable even in the presence of genetic alterations [6].

### 1.1. Defining Cancer Reversion: Critical Distinctions

We define cancer reversion as stable, heritable phenotypic normalization toward a non-malignant state that persists for extended periods (weeks to months in vitro; months to years in vivo) after treatment withdrawal. This distinguishes true reversion from related but distinct phenomena:

1. Stable Reversion (Durable Normalization): Sustained differentiation marker expression (>4 weeks in vitro, >3 months in vivo post-treatment); restored tissue architecture; normalized proliferation (<10% baseline); epigenetic consolidation (DNA methylation changes); functional restoration. Example: ATRA-treated APL cells undergo terminal differentiation into functional neutrophils that maintain phenotype indefinitely after treatment cessation.
2. Stimulus-Dependent Plasticity (Reversible Transition): Rapid reversion to malignancy (<2 weeks) upon treatment withdrawal; transient histone modifications without DNA methylation changes; retained tumor-initiating capacity; loss of organized architecture. Example: Breast cancer cells in 3D ECM scaffolds form organized structures but resume malignant growth within 7–14 days when returned to standard culture.
3. Dormancy/Quiescence: Cell cycle arrest (G0/G1) without differentiation; maintained stemness markers (Oct4, Sox2, Nanog); rapid growth resumption (48–72 h) when conditions permit. Not reversion—temporary growth suppression.
4. Senescence: Irreversible cell cycle arrest with SASP but no normalization; persistent DNA damage markers, p16/p21 upregulation, SA-β-gal positivity.
5. Cytotoxic Response: Cell death (apoptosis, necrosis, pyroptosis)—elimination, not reprogramming.

Throughout this review, we critically evaluate whether published examples represent stable reversion (category 1) versus alternative phenomena, assessing: duration of post-treatment stability, epigenetic consolidation, functional restoration, loss of tumor-initiating capacity, and terminal differentiation markers. This framework enables rigorous evaluation of reversion claims and identifies gaps regarding phenotypic stability.

### 1.2. Biological Foundations

The foundations for cancer reversion therapy stem from several key observations across different domains of cancer biology. First, the pioneering work of Takahashi and Yamanaka on cellular reprogramming demonstrated that differentiated cells could be reverted to a pluripotent state through the introduction of specific transcription factors [7]. This discovery illuminated the remarkable plasticity of cellular identity and suggested that cancer cells, too, might be amenable to reprogramming [8]. Second, rare but well-documented cases of spontaneous cancer regression in clinical settings provide compelling evidence that natural reversion processes exist [9,10,11]. Some of these spontaneous cancer regressions have been attributed to exposure to certain infectious toxins or antigens [12,13,14]. No doubt activated immune system has been implicated in this pathogenesis [15]. Third, experimental models have shown that manipulating the tumor microenvironment can suppress malignant behavior, indicating that cancer is not solely determined by intrinsic cellular properties but is significantly influenced by external cues [5,16,17].

The concept of cancer reversion also draws support from developmental biology, where normal regulatory mechanisms strictly control cell proliferation, differentiation, and tissue architecture. Cancer can be viewed as a disease of deregulated development, where cells have escaped these controls [18]. Reversion therapy aims to reactivate or re-impose these developmental constraints through stable epigenetic and transcriptional reprogramming, guiding cancer cells back toward normal behavior. This perspective has been reinforced by studies showing that embryonic environments can suppress the malignant phenotype of various cancer cells [19,20], suggesting that powerful normalization signals exist in development.

From a molecular perspective, cancer reversion therapy targets multiple layers of dysregulation characteristic of cancer cells [21]. At the genetic level, advances in gene editing technologies offer the potential to correct oncogenic mutations [22,23,24]. At the epigenetic level, modifying histone modifications, DNA methylation patterns, and chromatin structure may reactivate silenced tumor suppressor genes or silence hyperactive oncogenes [25,26,27]. At the signaling level, normalizing dysregulated pathways can restore appropriate responses to growth factors, differentiation signals, and apoptotic stimuli [28]. Each of these approaches aims not to kill cancer cells but to redirect them toward normal function.

The clinical relevance of cancer reversion therapy has already been demonstrated in certain contexts. The most notable success story is the treatment of APL with ATRA, which induces terminal differentiation of leukemic cells and leads to remarkably high cure rates [29,30]. Critically, this represents true stable reversion: differentiated APL cells maintain their mature neutrophil phenotype indefinitely, do not revert to blast crisis after ATRA withdrawal, and demonstrate functional maturation with phagocytic capacity, meeting all criteria for durable phenotypic normalization. This clinical success provides proof-of-principle that cancer reversion strategies can translate into effective therapies. More recently, studies using small-molecule inhibitors to target specific oncogenic drivers have shown the potential to induce differentiation in various cancer types [31,32], though many examples require critical evaluation regarding stability of the induced phenotypic changes.

Current evidence for cancer reversion comes from multiple experimental approaches. Some studies have demonstrated that modifying the ECM could normalize breast cancer cells in 3D culture systems [33,34]. However, many of these examples represent stimulus-dependent plasticity rather than stable reversion, as phenotypic normalization is rapidly lost upon return to standard culture conditions. Some recent studies have established support that microRNAs could reprogram cancer cells toward less aggressive states [35,36]. Work by Massagué and others revealed that targeting specific metastasis-promoting pathways could revert the metastatic phenotype [37,38]; the durability of these changes requires further validation in long-term studies. These findings collectively highlight the feasibility of cancer reversion as a therapeutic approach, though critical questions remain regarding which interventions produce truly stable versus reversible phenotypic changes.

### 1.3. Review Rationale and Objectives

This narrative review addresses the critical need for alternative cancer treatment paradigms that move beyond the limitations of conventional cytotoxic approaches. The rationale stems from mounting evidence that cancer cells retain a degree of plasticity that can be therapeutically exploited to revert them to non-malignant phenotypes, potentially circumventing issues of treatment resistance and severe side effects associated with traditional therapies. The novelty of this review lies in its comprehensive synthesis of disparate strands of research, from cellular reprogramming to tumor microenvironment modulation, epigenetic regulation to developmental biology, to present cancer reversion as a cohesive therapeutic framework rather than isolated experimental observations. Importantly, we critically distinguish between stable reversion and transient plasticity, providing a framework for evaluating the durability and clinical relevance of reported reversion phenomena.

Our aim is to evaluate the current state of cancer reversion research, critically analyzing both successful applications and persistent challenges, while highlighting emerging technologies that may accelerate progress in this field. The specific objectives are to:

(1) Establish clear definitions and criteria for stable reversion versus related phenomena (stimulus-dependent plasticity, dormancy, senescence); (2) Establish the theoretical foundations and biological plausibility of cancer reversion; (3) Examine current experimental and clinical evidence supporting stable reversion approaches, critically evaluating phenotypic durability; (4) Identify technological innovations with relevance to reversion strategies; (5) Outline promising future directions and critical obstacles that must be overcome to translate cancer reversion therapy from concept to clinical reality.

Through this systematic examination, we seek to stimulate interdisciplinary collaboration and accelerate the development of therapeutic strategies that reprogram rather than destroy cancer cells.

## 2. Methods

### 2.1. Search Strategy and Selection Criteria

This narrative review synthesizes current evidence, emerging technologies, and future directions in cancer reversion therapy. We conducted a comprehensive literature search using PubMed, Web of Science, and Scopus databases to identify relevant peer-reviewed articles published between January 1997 and August 2025. The 28-year timeframe was selected to capture seminal early work on cellular reprogramming while providing comprehensive coverage of recent advances.

The search strategy employed a systematic Boolean approach combining the following term groups:

Primary search terms: “cancer reversion” OR “tumor reprogramming” OR “phenotypic normalization” OR “malignant-to-benign transition”.

Secondary search terms (combined with AND operator): “differentiation therapy” OR “epigenetic reprogramming” OR “microenvironmental modulation” OR “oncogene addiction” OR “cellular plasticity” OR “cancer cell normalization”.

Tertiary search terms (for specificity): “cancer” OR “neoplasm” OR “malignancy” OR “tumor” OR “carcinoma” OR “sarcoma” OR “leukemia” OR “lymphoma”.

Specific cancer types (targeted searches): “acute promyelocytic leukemia,” “chronic myeloid leukemia,” “breast cancer,” “colorectal cancer,” “melanoma,” “neuroblastoma,” “glioblastoma,” “pancreatic cancer”.

Technology-specific terms: “CRISPR,” “single-cell,” “organoid,” “artificial intelligence,” “machine learning,” “spatial transcriptomics,” “epigenome editing”.

We performed initial broad searches (e.g., “cancer reversion” OR “tumor reprogramming”), followed by refined searches combining primary and secondary terms to focus on therapeutic approaches. Abstract screening was conducted, with full-text review of papers that directly addressed reversion mechanisms, experimental evidence, or clinical applications.

We supplemented the database searches with manual review of reference lists from key articles and recent reviews to identify additional relevant studies (“snowball” method). We also searched for ongoing clinical trials related to cancer reversion approaches using ClinicalTrials.gov and the World Health Organization’s International Clinical Trials Registry Platform (WHO ICTRP) (identifying relevant trials, of which specifically employed reversion-based endpoints).

### 2.2. Inclusion and Exclusion Criteria

We included original research articles, systematic reviews, meta-analyses, perspectives, and commentaries published in English that addressed molecular mechanisms, experimental evidence, clinical applications, or technological innovations related to cancer reversion. Studies were selected based on their relevance to understanding or inducing the transition of cancer cells toward less malignant or normal phenotypes.

Specific inclusion criteria:

- Studies demonstrating phenotypic normalization of cancer cells (in vitro, in vivo, or clinical)
- Research examining mechanisms of differentiation, epigenetic reprogramming, or microenvironmental normalization
- Clinical trials employing differentiation-inducing agents or reversion-based therapies
- Studies of spontaneous cancer regression with mechanistic investigation
- Technological innovations applicable to reversion therapy (single-cell analyses, CRISPR, organoids, AI/ML)
- Articles providing data on stability or reversibility of induced phenotypic changes

We excluded articles focusing solely on conventional cytotoxic therapies without reversion components, studies primarily addressing cancer prevention rather than treatment, and publications lacking peer review. Case reports were included only when they provided substantial mechanistic insights into spontaneous regression or treatment-induced reversion or when they documented long-term stability of phenotypic changes (>12 months follow-up).

Specific exclusion criteria:

- Studies reporting only growth inhibition or cytotoxicity without evidence of phenotypic normalization
- Papers using “reversion” terminology for genetic back-mutation or revertant cell lines
- Abstracts, conference proceedings, or non-English publications (except when containing critical data unavailable elsewhere)
- Studies with inadequate characterization of phenotypic changes (e.g., proliferation assays alone without differentiation markers).

### 2.3. Data Extraction and Synthesis

From selected articles, we extracted information regarding:

1. Conceptual frameworks and theoretical models of cancer reversion
2. Experimental evidence for reversion mechanisms (with specific attention to phenotypic stability data)
3. Clinical applications and outcomes of reversion-based approaches
4. Technological innovations with relevance to cancer reversion
5. Challenges, limitations, and future directions
6. Evidence characterizing stability vs. reversibility of phenotypic changes:
  - Duration of phenotypic maintenance after treatment withdrawal
  - Epigenetic consolidation markers (DNA methylation, histone modifications)
  - Functional assays (tumor-initiation capacity, metastatic potential)
  - Serial transplantation or long-term culture experiments

The extracted data were organized thematically rather than chronologically, focusing on four major approaches to cancer reversion: epigenetic reprogramming, microenvironmental modulation, differentiation therapy, and targeting oncogene addiction. For each approach, we synthesized evidence regarding mechanisms, experimental models, clinical translation, challenges, and future directions. For each cited example, we critically evaluated whether published data support stable reversion, stimulus-dependent plasticity, or alternative mechanisms based on the criteria outlined in Section 1.1.

Additionally, we analyzed emerging technologies with relevance to cancer reversion, including single-cell technologies, CRISPR-based approaches, organoid models, and artificial intelligence/machine learning applications. For each technology, we assessed current capabilities, limitations, and potential future applications in cancer reversion research and therapy.

### 2.4. Quality Assessment and Evidence Synthesis

For experimental studies, we assessed the rigor of methodology, reproducibility of findings, and biological relevance of models used. Specific quality criteria included:

- Sample size adequacy
- Appropriate controls (untreated, vehicle, or alternative treatment)
- Blinding and randomization (for animal studies)
- Validation across multiple cell lines or patient samples
- Independent replication by other research groups
- Assessment of phenotypic stability.

For clinical studies, we considered study design, sample size, outcome measures, and potential confounding factors. We prioritized:

- Randomized controlled trials over single-arm studies
- Studies with ≥20 patients (for early phase) or ≥100 patients (for late phase)
- Use of validated response criteria or reversion-specific endpoints
- Adequate follow-up duration (minimum 6 months for hematological malignancies, 12 months for solid tumors)
- Clear documentation of treatment duration and post-treatment observation periods.

We prioritized findings that had been independently validated across multiple studies or research groups.

For assessing evidence of stable vs. transient reversion, we evaluated:

- Level 1 evidence (strongest): Serial transplantation studies showing loss of tumor-initiating capacity; long-term clinical remissions (>2 years) maintained after treatment cessation; stable epigenetic changes persisting >3 months post-treatment
- Level 2 evidence (moderate): In vivo studies with ≥4 weeks post-treatment follow-up showing maintained phenotype; clinical responses lasting >6 months after treatment withdrawal; DNA methylation or stable histone modification changes
- Level 3 evidence (limited): In vitro studies with 2–4 weeks post-treatment observation; transient expression of differentiation markers; clinical benefit requiring continuous treatment
- Insufficient evidence: Studies without post-treatment follow-up; phenotypic assessment only during treatment exposure; lack of functional validation.

Given the narrative nature of this review, we did not employ formal meta-analysis or systematic review methodologies. Instead, we focused on providing a comprehensive and integrated perspective on the field, highlighting both consensus views and areas of ongoing debate or uncertainty. We explicitly note instances where reported “reversion” may represent alternative phenomena (plasticity, dormancy, cytotoxicity) and identify where additional validation is needed.

## 3. Understanding Cancer Reversion

Cancer reversion represents a fundamental reconceptualization of cancer therapy that challenges traditional treatment paradigms. At its core, stable cancer reversion refers to the process by which malignant cells transition back to a non-malignant state, regaining the characteristics of normal cells and maintaining this phenotype for extended periods even in the absence of continued therapeutic intervention, despite retaining their genetic alterations. This concept stands in stark contrast to conventional cancer treatments that primarily aim to eliminate cancer cells through surgery, radiation, or cytotoxic agents.

The theoretical underpinnings of cancer reversion emerge from a growing body of evidence suggesting that the cancer phenotype is not irreversibly determined by genetic mutations alone. Rather, cancer can be viewed as a dynamic cellular state that may, under specific conditions, be redirected toward normalcy [5]. This plasticity of the cancer phenotype opens therapeutic windows that previous approaches overlooked.

One of the pivotal discoveries supporting the concept of cancer reversion came from the groundbreaking work of Takahashi and Yamanaka, who demonstrated that differentiated somatic cells could be reprogrammed into iPSCs through the introduction of just four transcription factors (named Myc, Oct3/4, Sox2 and Klf4) [7]. This remarkable finding revealed an unexpected degree of cellular plasticity and suggested that even cells with firmly established identities, including cancer cells, might be amenable to reprogramming through the modulation of key regulatory factors. Subsequent research has shown that similar reprogramming approaches can indeed alter the behavior of cancer cells, driving them toward less aggressive phenotypes [35,36]. However, the stability of these induced changes varies considerably, with some representing true reversion and others reflecting transient plasticity.

Further evidence for the feasibility of cancer reversion comes from clinical observations of spontaneous regression, wherein cancers are resolved without therapeutic intervention. Though rare, these cases have been documented across multiple cancer types including melanoma, neuroblastoma, and certain lymphomas [39,40,41]. Detailed analysis of these cases reveals that such regressions often coincide with significant changes in the host environment, such as infection, pregnancy, or trauma, suggesting that powerful extrinsic signals can trigger reversion processes. For instance, Everson and Cole as far back as 1956 reviewed published case reports of spontaneous cancer regressions and suggested that these cases may be associated with specific immune signatures, indicating potential mechanistic pathways that might be therapeutically exploitable [42]. Importantly, documented spontaneous regressions often represent stable, durable responses persisting for years without recurrence, providing proof-of-concept for therapeutically induced stable reversion.

The influential work of Weigelt, Bissell and colleagues has provided compelling experimental evidence that the microenvironment plays a crucial role in determining whether cells express normal or malignant phenotypes. Their landmark studies demonstrated that malignant breast cancer cells could revert to normal-appearing structures when cultured in 3D matrices that mimicked the normal breast microenvironment [33,43]. Remarkably, these reverted structures displayed normalized growth patterns and restored tissue architecture despite retaining their genetic mutations. However, critical evaluation reveals that many of these “reverted” cells rapidly return to malignant phenotypes when removed from the normalizing microenvironment (within 7–14 days), indicating stimulus-dependent plasticity rather than stable reversion. The distinction is therapeutically crucial: stable reversion would permit time-limited treatment, whereas stimulus-dependent plasticity requires continuous therapeutic intervention to maintain the normalized state.

Epigenetic mechanisms represent another critical dimension of cancer reversion. Cancer cells typically exhibit profound epigenetic dysregulation, including aberrant DNA methylation patterns, histone modifications, and altered chromatin structure. Studies by Jones and Baylin have shown that reversing these epigenetic abnormalities can restore normal gene expression patterns and cellular behavior [44,45]. For example, treatment with DNMT inhibitors has been shown to reactivate silenced tumor suppressor genes and induce differentiation in certain hematological malignancies, effectively reversing aspects of the cancer phenotype [46,47]. The durability of these changes correlates with the extent of DNA methylation remodeling: transient histone acetylation produces reversible effects, whereas stable DNA demethylation can produce lasting phenotypic normalization persisting for months after treatment cessation.

The developmental biology perspective provides additional support for cancer reversion. During normal development, cells navigate complex transitions between proliferation, differentiation, and tissue organization under tight regulatory control. Cancer can be viewed as a state where cells have escaped these developmental constraints. Studies examining the interactions between cancer cells and embryonic environments reveal that developmental signals can override malignant programming [19,20]. Hendrix and colleagues demonstrated that aggressive melanoma cells adopted more benign characteristics when exposed to embryonic zebrafish or chick microenvironments, underscoring the power of developmental contexts to normalize cancer cells [48]. These studies demonstrate that embryonic microenvironments can induce phenotypic normalization, though the stability of these changes upon return to adult microenvironments remains incompletely characterized.

Mechanistically, cancer reversion operates through multiple interconnected pathways. At the cellular level, reversion involves changes in proliferation, differentiation, migration, and cell survival. These changes are orchestrated by alterations in signaling networks, metabolic activities, and gene expression programs. Key pathways implicated in reversion include Wnt/β-catenin, Notch, Hedgehog, TGF-β, and various receptor tyrosine kinase cascades, which collectively regulate cell fate decisions and tissue architecture [49].

Understanding the molecular switches that control transitions between malignant and non-malignant states has become a central focus of cancer reversion research. These include master transcription factors, microRNAs, long non-coding RNAs, and chromatin modifiers that orchestrate broad transcriptional programs. For instance, some studies identified specific microRNAs capable of suppressing breast cancer metastasis by regulating multiple target genes simultaneously [50,51]. Similarly, transcription factors such as GATA3, ESR1, FOXM1 and FOXA1 have been shown to promote differentiation and suppress malignant traits in breast cancer models [52].

The therapeutic Implications of cancer reversion extend beyond academic Interest. Several clinically approved therapies already operate partly through reversion mechanisms, although they were not initially conceptualized in these terms. The success of ATRA in APL represents perhaps the clearest example of differentiation therapy, where malignant promyelocytes are induced to mature into functional neutrophils [29,30]. This represents true stable reversion: patients achieve molecular remission that persists for years after treatment completion, with differentiated cells demonstrating terminal maturation and no capacity for dedifferentiation. Similarly, the efficacy of imatinib in CML likely involves not only inhibition of BCR-ABL kinase activity but also restoration of normal hematopoietic differentiation pathways [53], though a subset of patients experience disease relapse after treatment discontinuation, suggesting incomplete reversion in some cases.

As our understanding of cancer reversion deepens, it becomes increasingly clear that this approach offers unique advantages over traditional cancer therapies. By restoring normal cellular behavior rather than inducing cell death, reversion therapies may produce fewer toxic side effects and potentially overcome resistance mechanisms that typically emerge under selective pressure from cytotoxic treatments. Furthermore, targeting the fundamental properties that distinguish cancer cells from normal cells, such as differentiation state, tissue organization, and microenvironmental interactions, may address the root causes of malignancy rather than merely its manifestations. However, realizing these benefits requires distinguishing therapeutic strategies that produce stable, durable reversion from those that induce only transient plasticity, necessitating continued therapeutic pressure to maintain the normalized phenotype.

Table 1 presents a comparative analysis of predominant reversion strategies across major cancer types, highlighting how different malignancies may require tailored approaches based on their underlying biology and identifying the current clinical development status for each strategy. For each strategy, we indicate the level of evidence for stable vs. stimulus-dependent responses where such data are available.

Stability Evidence Levels:

- Level 1 (Strongest): Serial transplantation showing loss of tumor-initiating capacity; clinical remissions >2 years maintained after treatment cessation; stable epigenetic changes >3 months post-treatment.
- Level 2 (Moderate): In vivo studies with ≥4 weeks post-treatment follow-up; clinical responses >6 months after treatment withdrawal; DNA methylation or stable histone modifications.
- Level 3 (Limited): In vitro studies with 2–4 weeks observation; phenotypic changes during treatment; limited post-treatment data.
- Insufficient: Studies without post-treatment follow-up; assessment only during treatment exposure.

## 4. Current Evidence and Approaches

**Figure 1:**
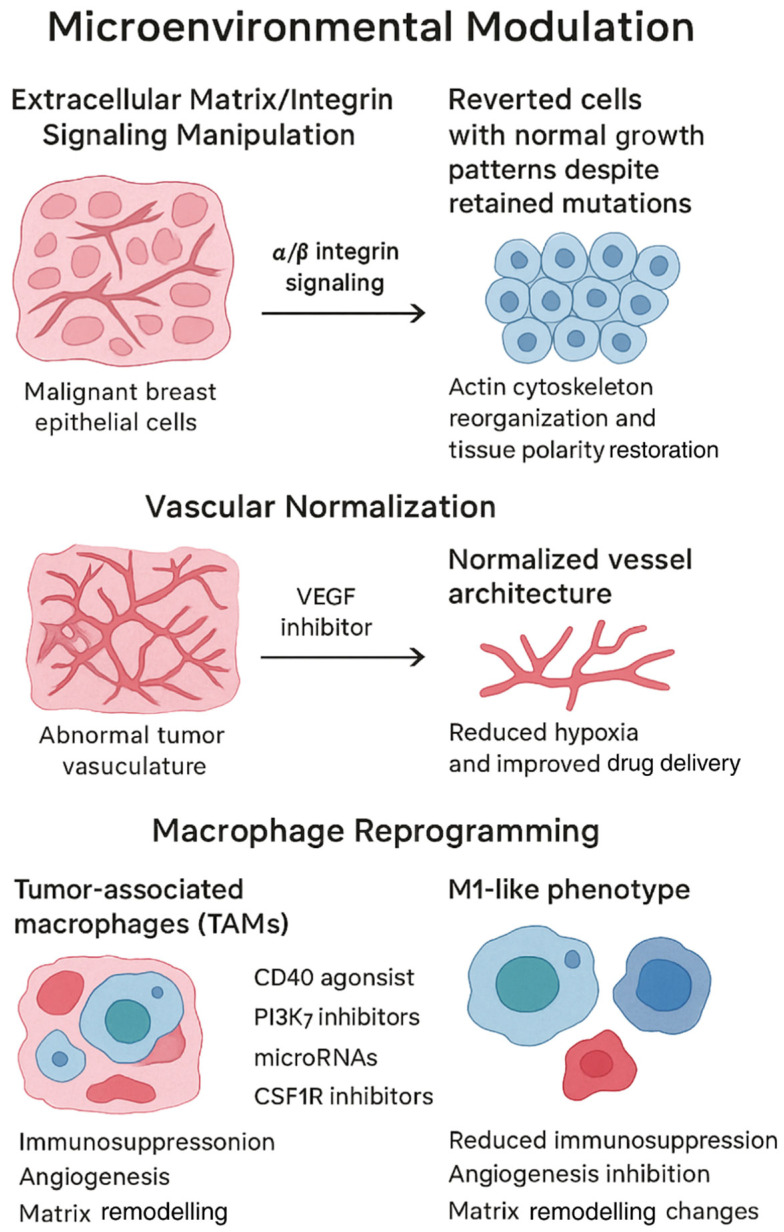
Microenvironmental modulation strategies in cancer reversion. (1) Extracellular matrix/integrin signaling manipulation altering α/β integrin signaling reorganizes the actin cytoskeleton and restores polarity, reverting malignant breast epithelial cells to normal phenotypes. (2) Vascular normalization: VEGF inhibitors remodel chaotic tumor vasculature to improve perfusion, reduce hypoxia, and enhance therapy response. (3) Macrophage reprogramming: tumor-associated macrophages are shifted from an M2 tumor-promoting state to an M1 anti-tumor phenotype using agents like CD40 agonists, PI3Kγ inhibitors, microRNAs, and CSF1R inhibitors.

Figure 1 illustrates three microenvironmental modulation strategies, ECM/integrin manipulation normalizing breast cancer architecture, VEGF inhibition producing vascular normalization, and macrophage reprogramming from M2 to M1 phenotype, emphasizing that all three approaches produce context-dependent, stimulus-dependent plasticity rather than stable reversion, as phenotypic normalization is rapidly lost (within days to weeks) upon treatment withdrawal or removal from normalizing microenvironments, necessitating continuous therapeutic intervention or combination with agents that consolidate differentiated states.

### 4.1. Epigenetic Reprogramming

Epigenetic modifications represent a layer of regulation that controls gene expression without altering the DNA sequence itself. In cancer, epigenetic dysregulation contributes significantly to disease progression by silencing tumor suppressor genes and activating oncogenes. This understanding has spurred research into epigenetic reprogramming as a strategy for cancer reversion, with substantial evidence supporting its therapeutic potential. Importantly, the durability of epigenetic reprogramming-induced phenotypic changes depends critically on whether transient histone modifications or stable DNA methylation alterations are achieved.

Marks et al. [73] provided a comprehensive framework for targeting cancer epigenetics, highlighting HDAC inhibitors as particularly promising agents. Their work demonstrated that HDAC inhibitors can restore acetylation patterns on histones, leading to reactivation of silenced genes involved in differentiation and cell cycle control. In AML, for instance, HDAC inhibitors have been shown to induce differentiation of leukemic blasts into functional mature cells [74,75]. However, histone acetylation is a dynamic, reversible modification: phenotypic changes induced by HDAC inhibitors often regress within 2–4 weeks after treatment withdrawal, indicating stimulus-dependent plasticity rather than stable reversion in many cases. The FDA approval of several HDAC inhibitors, including vorinostat and romidepsin for certain lymphomas after successful trials [76,77,78,79], represents clinical validation of this approach, though these agents typically require continuous or repeated administration to maintain clinical benefit, consistent with reversible epigenetic effects.

DNA methylation, another key epigenetic mechanism, often becomes aberrant in cancer cells, with hypermethylation of promoter regions silencing critical tumor suppressor genes. Goffin and Eisenhauer [80] demonstrated that DNMT inhibitors such as 5-azacytidine and decitabine can reverse these methylation patterns, reactivating silenced genes and restoring normal cellular functions. In myelodysplastic syndrome and certain leukemias, these agents have shown clinical efficacy by promoting cellular differentiation and reducing malignant potential. Importantly, DNA methylation changes are more stable than histone modifications: their effects can persist for weeks to months after treatment cessation in responsive cells, suggesting greater potential for durable reversion [46,47]. However, even with DNMT inhibitors, maintenance therapy is often required to sustain clinical responses, indicating that complete, irreversible reversion remains challenging to achieve.

More recently, inhibitors targeting BET proteins have emerged as powerful tools for epigenetic reprogramming. Delmore and colleagues [81] demonstrated that BET inhibitors could disrupt the interaction between bromodomain proteins and acetylated histones, effectively blocking aberrant gene expression in MYC-driven cancers. In MYC-amplified cancer models, BET inhibition downregulated MYC-dependent transcription programs and induced differentiation and apoptosis [81,82]. These MYC-amplified cancer models include various cancer types, such as leukemia, lymphoma, and certain solid tumors, highlighting the broad applicability of this approach. Similar to HDAC inhibitors, BET inhibitors produce primarily reversible effects: phenotypic normalization is typically lost within days to weeks after treatment withdrawal, necessitating continuous therapeutic exposure.

### 4.2. Microenvironmental Modulation

The tumor microenvironment profoundly influences cancer cell behavior through complex interactions involving ECM, stromal cells, immune cells, and signaling molecules. Targeting these interactions offers unique opportunities for cancer reversion therapy by restoring the regulatory influences that maintain normal tissue architecture and function. However, critical evaluation reveals that many microenvironment-based normalization strategies produce stimulus-dependent plasticity rather than stable reversion, as phenotypic normalization is rapidly lost when cells are removed from the normalizing microenvironment.

Groundbreaking works published by scientists in the last three decades changed our understanding of the role of the microenvironment in cancer biology. Abu-Tayeh et al. [64] and Weaver et al. [65] demonstrated that malignant breast epithelial cells could be reverted to a normal phenotype by manipulating the ECM and integrin signaling. Using 3D culture systems that mimic the normal breast tissue architecture, they showed that altering alpha and beta integrin signaling led to reorganization of the actin cytoskeleton, restoration of tissue polarity, and normalization of cell growth patterns. These reverted cells retained their genetic mutations but no longer exhibited malignant behavior [83,84], providing compelling evidence that phenotype can override genotype under appropriate microenvironmental conditions. However, subsequent studies have demonstrated that this normalization is highly context-dependent: when “reverted” cells are removed from the 3D ECM scaffold and returned to standard 2D culture or injected into permissive in vivo environments, they rapidly (within 7–14 days) resume malignant growth patterns, anchorage-independent growth, and invasive behavior. This indicates stimulus-dependent plasticity rather than stable reversion. The therapeutic implication is significant: achieving durable clinical benefit would require either continuous maintenance of the normalizing microenvironment (impractical) or identification of interventions that consolidate the normalized phenotype through irreversible epigenetic or differentiation programs.

Abnormal tumor vasculature represents another microenvironmental factor that promotes cancer progression. Jain [85,86] pioneered the concept of vascular normalization through VEGF inhibitors as a therapeutic strategy. Rather than eliminating tumor blood vessels, vascular normalization aims to restore a more normal vascular architecture, improving blood flow, reducing hypoxia, and enhancing drug delivery. This approach has shown clinical benefits in combination with chemotherapy or immunotherapy across multiple cancer types. By alleviating hypoxia, vascular normalization also reduces the activation of hypoxia-inducible factors that drive aggressive cancer phenotypes [87], potentially contributing to phenotypic normalization. However, vascular normalization is a dynamic, stimulus-dependent process: tumor vasculature typically reverts to its abnormal state within 1–3 weeks after VEGF inhibitor withdrawal, followed by rebound hypoxia and reactivation of aggressive cancer phenotypes. Thus, vascular normalization represents a therapeutic strategy that improves drug delivery and transiently suppresses aggressive phenotypes, but does not constitute stable cancer cell reversion. Clinical benefit requires continuous VEGF inhibition, and resistance mechanisms eventually emerge even with sustained treatment.

TAMs typically adopt an M2-like phenotype that promotes cancer progression through immunosuppression, angiogenesis, and matrix remodeling. Su et al. [88] demonstrated that these macrophages could be reprogrammed toward an M1-like phenotype with anti-tumor properties. Various approaches have shown promise in this regard, including CD40 agonists [89,90], PI3Kγ inhibitors [91], microRNAs [88], and CSF1R inhibitors [92]. In preclinical models, macrophage reprogramming reduced tumor growth and metastasis while enhancing responses to other therapies [93,94,95]. Clinical trials exploring this strategy have reported encouraging results [96,97], suggesting that reshaping the immune microenvironment may contribute to cancer phenotypic normalization. However, macrophage phenotypes are highly plastic and responsive to local cytokine milieus: M1-polarized macrophages revert to M2-like phenotypes within days when the polarizing stimulus is withdrawn or when tumor-derived factors (e.g., CSF-1, IL-10, TGF-β) are present. Therefore, macrophage reprogramming represents a component of combination therapy strategies that must be sustained to maintain anti-tumor effects, rather than a standalone reversion approach that produces durable cancer cell normalization.

### 4.3. Differentiation Therapy

Differentiation therapy exploits the principle that cancer often represents a state of blocked or aberrant differentiation. By inducing cancer cells to mature into more differentiated states, this approach aims to restore normal cellular functions and reduce malignant potential, effectively achieving cancer reversion through developmental reprogramming. Importantly, differentiation therapy encompasses a spectrum from reversible, transient differentiation to irreversible, terminal differentiation. Only the latter represents true stable reversion with potential for treatment-free remission.

The paradigmatic success story in differentiation therapy, and the gold standard for stable reversion, remains the treatment of APL with ATRA. Cicconi and colleagues [56] in an updated trial reported remarkable clinical outcomes with ATRA combined with arsenic trioxide, achieving complete remission rates exceeding 95% and high long-term survival rates. ATRA binds to the PML-RARα fusion protein characteristic of APL, relieving its repressive effect on differentiation genes and allowing promyelocytes to mature into functional neutrophils [57]. This therapy transformed APL from a highly lethal disease to one of the most curable forms of leukemia, providing undeniable proof-of-concept for differentiation-based cancer reversion. Critically, this represents true stable reversion meeting all criteria: (1) Terminal differentiation: APL cells progress through irreversible maturation stages to become functional neutrophils with phagocytic capacity, oxidative burst capability, and chemotactic responses; (2) Loss of self-renewal: Differentiated cells cannot dedifferentiate or maintain leukemic clone expansion; (3) Durable remissions: Many patients remain in molecular remission for >10–20 years after completing finite treatment courses (typically 6–12 months), without requiring maintenance therapy; (4) Functional restoration: Differentiated cells integrate into normal hematopoiesis, performing physiological neutrophil functions; (5) Epigenetic consolidation: Terminal differentiation is accompanied by stable chromatin remodeling and DNA methylation changes at myeloid differentiation loci.

The success of ATRA in APL provides a critical benchmark: true cancer reversion through differentiation therapy should produce irreversible maturation with loss of tumor-initiating capacity, sustained clinical benefit after treatment withdrawal, and functional restoration of normal cell activities. We evaluate other differentiation approaches against these criteria.

Vitamin D derivatives have shown potential in various cancer models but have not achieved the stable, durable reversion seen with ATRA in APL. Several studies have provided both preclinical and clinical evidence demonstrating that 1,25-dihydroxyvitamin D3 and its analogs can induce differentiation, inhibit proliferation and angiogenesis, and promote apoptosis in multiple cancer types, including lung, prostate, and colorectal cancers [98,99,100,101,102,103,104,105,106]. The mechanism responsible for the induction of differentiation is mediated through VDR-dependent gene expression [107]. Inhibition of proliferation occurs via cell cycle arrest at the G0/G1 phase, which is facilitated by the upregulation of cyclin-dependent kinase inhibitors p21 and p27, as well as the inhibition of cyclin D1 [108,109]. To inhibit angiogenesis, D3 downregulates VEGF expression and impedes HIF signaling [110,111]. Regarding apoptosis, D3 promotes cell death through activation of mitochondrial apoptotic mechanisms, such as the caspase cascade, which leads to the downregulation of anti-apoptotic proteins [112,113]. However, several key limitations distinguish vitamin D effects from true stable reversion: (1) Differentiation is typically incomplete, with cells retaining proliferative capacity and tumor-forming potential; (2) Effects are largely reversible, tumor regrowth occurs rapidly after vitamin D withdrawal in most models; (3) Growth inhibition (cytostatic effects) rather than terminal differentiation accounts for much of the anti-tumor activity; (4) Clinical translation has been hampered by hypercalcemia at therapeutic doses, and newer vitamin D analogs with reduced calcemic effects are showing promise in early-phase clinical trials [114] but have not yet demonstrated durable remissions after treatment cessation. Thus, vitamin D derivatives induce partial, reversible differentiation and growth arrest rather than the stable reversion exemplified by ATRA in APL.

**Figure 2:**
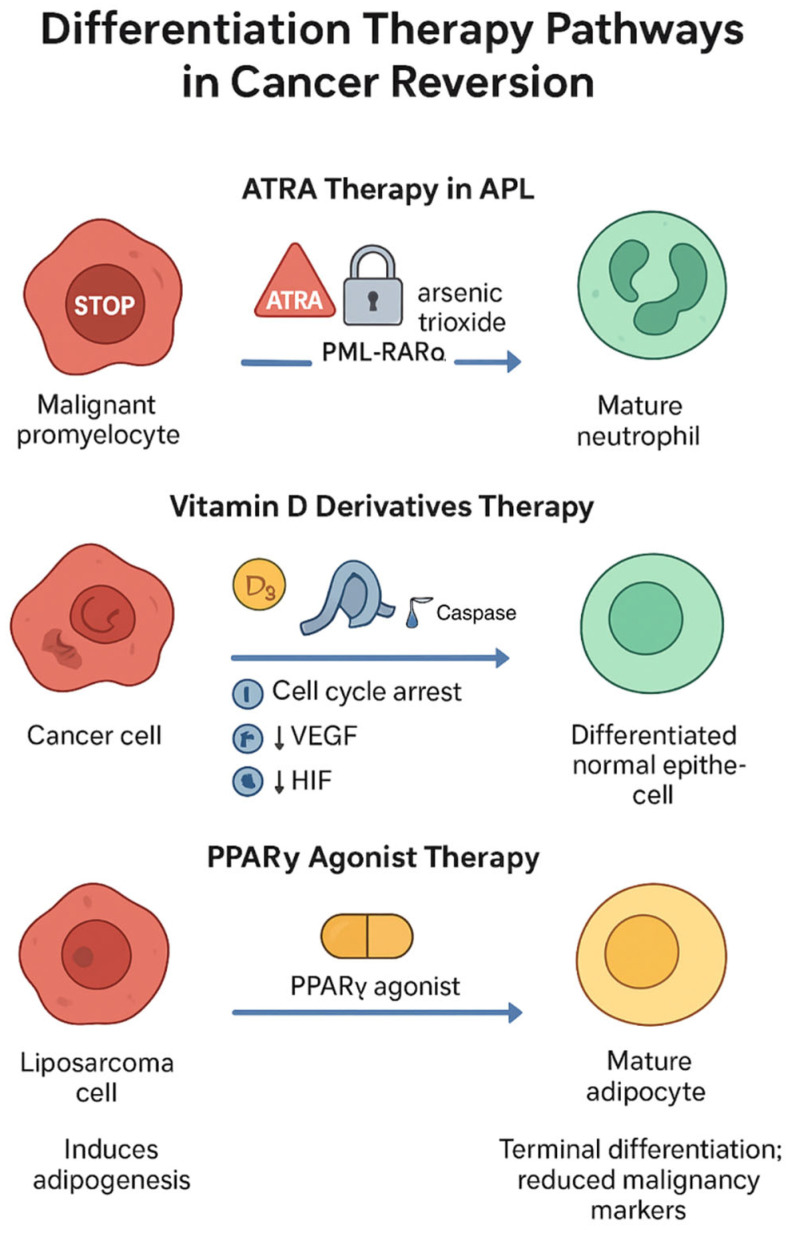
Differentiation therapy pathways in cancer reversion. Panel 1 shows ATRA + arsenic trioxide overcoming PML-RARα repression to restore myeloid maturation in APL. Panel 2 illustrates how vitamin D3 binding to VDR induces cell cycle arrest, inhibits VEGF/HIF signaling, and promotes apoptosis, leading to normal epithelial differentiation. Panel 3 depicts PPARγ agonists driving adipogenic differentiation in liposarcoma, resulting in terminally differentiated adipocytes with reduced malignancy markers.

PPARγ represents another differentiation target with proven clinical relevance but mixed evidence for stable vs. reversible effects. Tontonoz and colleagues [115] demonstrated that PPARγ agonists such as pioglitazone could induce terminal differentiation in liposarcoma cells, transforming malignant cells into mature adipocytes. This finding was confirmed by Demetri et al. [116] on three patients with liposarcoma using another PPARγ agonist called troglitazone, where tumor biopsies revealed induction of differentiation markers and reduced proliferation. In these cases, there was evidence of morphological maturation toward adipocyte-like cells with lipid accumulation, suggesting potentially irreversible differentiation. However, long-term follow-up data on phenotypic stability after drug withdrawal were not available to confirm true stable reversion. Although troglitazone was later withdrawn due to hepatotoxicity [117] and rosiglitazone, another PPARγ agonist, was not effective as an anti-tumor drug in the treatment of liposarcoma [118]. Nevertheless, newer PPARγ agonists continue to be investigated for their differentiation-inducing properties in various malignancies. Efatutazone, a new PPARγ agonist, showed strong differentiation-inducing potential in a Phase I study [119]. PPARγ agonists have also performed well in combinations with other anti-cancer therapies [120,121]. Overall, PPARγ-mediated differentiation shows promise but lacks the extensive validation of long-term, treatment-free remissions that would establish it as producing stable reversion comparable to ATRA in APL. Current evidence suggests effects may be partially reversible, requiring sustained treatment or combination approaches to maintain clinical benefit. Figure 2 provides a detailed pathway overview of differentiation therapy, visually mapping drug actions, molecular targets, and resulting cellular differentiation states.

### 4.4. Targeting Oncogene Addiction

Many cancers develop dependency on specific oncogenic drivers, a phenomenon termed “oncogene addiction.” Targeting these critical dependencies can not only inhibit proliferation but also release cells from oncogene-imposed developmental blocks, allowing restoration of normal differentiation programs and potentially achieving cancer reversion. However, whether oncogene inhibition produces stable reversion or reversible growth suppression varies considerably depending on the specific oncogenic driver, cancer context, and duration of oncogene inhibition.

The treatment of CML with the BCR-ABL tyrosine kinase inhibitor imatinib represents a landmark achievement in targeted cancer therapy and provides important insights into both the potential and limitations of oncogene inhibition as a reversion strategy. Piazza and colleagues [58] reported unprecedented clinical responses in CML patients treated with imatinib, with the majority achieving complete cytogenetic remission and fewer (15%) patients with relapse. Beyond simply inhibiting proliferation, imatinib appears to restore normal hematopoietic differentiation by removing the BCR-ABL-imposed block on differentiation pathways [59]. Critically, long-term follow-up has revealed heterogeneous outcomes regarding phenotypic stability [58]: (1) Treatment-Free Remission (TFR) subset (40–60% of patients with deep molecular remission): These patients maintain durable molecular remission for years after imatinib discontinuation, suggesting stable reversion with restoration of normal hematopoietic differentiation programs. This represents one of the few solid examples of stable reversion in solid/hematological malignancy achieved through targeted therapy. (2) Treatment-Dependent subset (40–60%): Molecular or cytogenetic relapse occurs within 6–12 months of imatinib cessation, indicating that BCR-ABL inhibition produces growth suppression and partial differentiation but not stable reversion in these patients. Continued treatment is required to maintain remission. (3) Leukemic stem cell persistence: Even in TFR patients, sensitive techniques can detect BCR-ABL + cells at low levels, suggesting that some cancer cells persist in a dormant or quiescent state rather than having undergone true reversion to normalcy. This heterogeneity indicates that oncogene inhibition can produce stable reversion in a subset of patients (likely depending on additional genetic/epigenetic factors), but does not universally achieve stable phenotypic normalization. The predictive factors determining who achieves TFR vs. requiring continuous therapy remain under investigation.

Similar principles apply to BRAF-mutant melanoma treated with BRAF inhibitors. Chapman and colleagues [122] demonstrated impressive response rates with the BRAF inhibitor vemurafenib in BRAF V600E-mutant melanoma. Alongside direct antiproliferative effects, BRAF inhibition induces changes in melanoma cell differentiation status, with increased expression of melanocyte differentiation antigens and restored pigmentation in some cases. However, these changes are typically transient and reversible: (1) Resistance development: Most patients develop resistance within 6–12 months despite continued BRAF inhibitor treatment, with tumors resuming aggressive growth through activation of bypass signaling pathways (NRAS, MEK, receptor tyrosine kinases); (2) Rapid relapse upon discontinuation: Cessation of BRAF inhibitors leads to rapid tumor progression (within weeks), indicating reversible growth suppression rather than stable reversion; (3) Incomplete differentiation: Even when melanocytic differentiation markers are upregulated, cells retain tumor-initiating capacity and invasive properties, indicating partial, reversible phenotypic changes. Although combination strategies targeting multiple nodes in the MAPK pathway have improved outcomes [123], resistance mechanisms eventually emerge, and durable remissions after treatment discontinuation are rare. Thus, BRAF inhibition in melanoma represents effective targeted therapy but not stable reversion in most cases.

The concept of “oncogene amnesia,” initially introduced by Bernard Weinstein, provides a mechanistic framework for understanding how oncogene inhibition can lead to cancer reversion. This concept provides an explanation of how oncogene activation drives tumorigenesis by inducing cellular amnesia that promotes unchecked proliferation and disables key checkpoints for cell death, renewal, and genomic stability [124]. To support this phenomenon using conditional transgenic models of MYC-driven tumors, Jain [125] demonstrated that even brief inactivation of the MYC oncogene could induce sustained tumor regression through differentiation and apoptosis. Importantly, upon MYC reactivation, some tumors failed to recur, suggesting that cells had entered a differentiation state that rendered them insensitive (“amnesic”) to the oncogenic signal, potentially representing true stable reversion. However, this was context-dependent: some tumors rapidly re-emerged upon MYC reactivation, indicating incomplete or reversible differentiation. These studies in genetically engineered mouse models provide proof-of-principle that transient oncogene inhibition can, in some contexts, induce stable phenotypic changes. However, translating this to human cancers remains challenging: human tumors typically have accumulated multiple genetic alterations beyond the primary oncogenic driver, and these secondary alterations may prevent stable reversion even when the primary oncogene is inhibited.

## 5. Emerging Technologies

Table 2 offers a detailed framework for monitoring therapeutic responses through various biomarker categories, addressing one of the major challenges in the field: how to reliably detect and quantify successful reversion in clinical settings. Critically, this table distinguishes biomarkers that can detect true stable reversion from those that may reflect transient plasticity or reversible phenotypic changes.

Interpretation Framework for Assessing Stability:

1. Stable Reversion (True Phenotypic Normalization): DNA methylation changes present; sustained differentiation marker expression >3 months post-treatment; loss of tumor-initiating capacity in serial transplantation; restoration of tissue architecture and normal functions; low Ki-67 with mature cell morphology.
2. Transient Plasticity (Stimulus-Dependent): Only histone modification changes (no DNA methylation); differentiation markers lost within weeks of treatment cessation; retained tumor-initiating capacity; rapid Ki-67 rebound upon treatment withdrawal.
3. Dormancy/Quiescence (Not Reversion): Low Ki-67 but retained stemness markers (Oct4, Sox2); no differentiation marker expression; no functional restoration; tumor-initiating capacity preserved; rapid proliferation resumption when conditions permit.
4. Senescence (Not Reversion): High p16/p21, SA-β-gal positive; permanent growth arrest but no differentiation; retained malignant molecular signature; SASP (inflammatory secretome).

Recommended Biomarker Panel for Clinical Assessment of Reversion:

- Minimal panel: Differentiation markers (IHC/flow) + Ki-67 + morphology (serial biopsies/imaging);
- Comprehensive panel: Above + cfDNA methylation (liquid biopsy) + metabolic imaging (FDG-PET) + functional assays (where feasible);
- Research/validation studies: Above + serial transplantation + single-cell epigenomics + clonal tracking.

### 5.1. Single-Cell Technologies

The advent of single-cell technologies has transformed our understanding of cancer as a heterogeneous disease composed of diverse cell populations with distinct molecular profiles and functional states. These technologies offer unprecedented insights into cancer cell plasticity and state transitions, providing critical information for developing effective reversion strategies. Importantly, single-cell analyses can distinguish stable cell state transitions from transient, reversible fluctuations by tracking individual cells over time and characterizing the epigenetic landscapes that determine phenotypic stability.

Single-cell RNA sequencing (scRNA-seq) has emerged as a powerful tool for mapping cellular heterogeneity within tumors. Patel and colleagues [138] applied scRNA-seq to primary glioblastoma samples, revealing remarkable intratumoral heterogeneity with individual cells exhibiting distinct transcriptional programs related to oncogenic signaling, proliferation, immune response, and hypoxia. Their work demonstrated that tumors contain cells at various points along a developmental continuum rather than discrete subtypes, suggesting potential opportunities for redirecting cancer cells toward less aggressive states. Subsequent studies have extended these findings across multiple cancer types, identifying rare cell populations that drive treatment resistance and metastasis. Tirosh et al. [139] used scRNA-seq to identify a small subpopulation of melanoma cells with stem-like properties that contribute to tumor relapses after treatment, providing potential targets for reversion-based interventions. Critically, when combined with longitudinal sampling, scRNA-seq can distinguish transient transcriptional responses from stable cell state transitions: cells that have undergone stable reversion show persistent transcriptional programs characteristic of differentiated states even weeks after treatment withdrawal, whereas transiently responding cells rapidly revert to undifferentiated transcriptional signatures.

Mass cytometry (CyTOF) complements transcriptomic approaches by enabling simultaneous measurement of dozens of protein markers at the single-cell level. Bendall and colleagues [140] pioneered this technology in hematology research, revealing previously unrecognized cellular subpopulations with distinct functional properties and developmental trajectories. By incorporating phospho-specific antibodies, CyTOF can map signaling network activities across thousands of individual cells, providing dynamic views of how cancer cells respond to therapeutic perturbations. This capability proves invaluable for identifying agents that shift cancer cells toward more differentiated states and, critically, for determining whether these shifts are sustained or reversible by measuring signaling pathway activities at multiple timepoints after treatment withdrawal. Levine et al. [141] used mass cytometry to characterize signaling responses in AML, identifying patient-specific vulnerabilities that could be exploited for personalized differentiation therapy.

Spatial transcriptomics adds another crucial dimension by preserving information about cellular location within tissues. Ståhl and colleagues [142] developed a method that combines histological imaging with spatially resolved RNA sequencing, allowing transcriptome analysis with preservation of tissue architecture. This technology is particularly relevant for assessing reversion: stable phenotypic normalization should manifest as restoration of normal tissue architecture with appropriately organized cell types, whereas transient plasticity may show normalized gene expression without proper spatial organization. In breast cancer studies, spatial transcriptomics has uncovered region-specific gene expression patterns associated with varying degrees of malignancy, highlighting the importance of spatial context in determining cancer cell behavior.

Integration of multiple single-cell modalities, genomics, transcriptomics, epigenomics, and proteomics, now enables comprehensive characterization of cancer cell states and the factors governing transitions between states. Recent technological advances, such as Parallel-seq, enable simultaneous analysis of chromatin accessibility and gene expression in single cells by jointly profiling scATAC-seq and scRNA-seq, thereby providing insights into epigenetic regulation [143]. These multi-modal approaches are critical for reversion research because they reveal whether observed phenotypic changes are accompanied by stable epigenetic alterations (suggesting durable reversion) or merely reflect transient transcriptional responses without chromatin remodeling (indicating reversible plasticity). Cells undergoing stable reversion show coordinated changes: closed chromatin at stemness loci, open chromatin at differentiation loci, sustained expression of lineage-specific genes, and loss of self-renewal signatures. In contrast, transiently responding cells show transcriptional changes without corresponding stable chromatin remodeling.

### 5.2. Crispr-Based Approaches

CRISPR-Cas9 technology has revolutionized genetic manipulation by providing unprecedented precision, efficiency, and versatility. In cancer reversion research, CRISPR-based approaches offer powerful tools for understanding the genetic and epigenetic determinants of cancer cell states and for developing targeted therapeutic strategies. Critically, CRISPR approaches can be used to test whether specific genetic or epigenetic alterations are necessary for maintaining malignant vs. differentiated states, thereby identifying intervention points for stable reversion.

Genome-wide CRISPR screens have emerged as a systematic approach to identify genes essential for maintaining cancer phenotypes. Wang and colleagues [144] conducted genome-scale CRISPR screens across multiple AML cell lines, identifying context-specific vulnerabilities and dependency networks. These screens have revealed genes whose disruption induces differentiation or reverts malignant phenotypes, providing potential targets for reversion therapy. For example, CRISPR screens in AML identified factors that maintain the undifferentiated state; their inhibition promotes differentiation and reduces leukemic potential [145]. Similar screens in solid tumors have uncovered therapeutic targets in cancers with few mutations, such as pediatric rhabdoid tumors, where receptor tyrosine kinases and the downstream effector SHP2 were identified as key dependencies despite the absence of recurrent genomic alterations [146]. Importantly, CRISPR screens can distinguish genes required for survival (whose knockout causes cell death) from genes required for maintaining undifferentiated state (whose knockout induces differentiation), the latter represent ideal reversion therapy targets.

Recent advances have moved beyond gene knockout to precise genetic correction. Anzalone and colleagues [147] developed prime editing, a versatile genome editing method that can directly correct a wide range of mutation types without requiring double-strand breaks or donor DNA templates. This technology offers the potential to precisely correct oncogenic driver mutations, addressing the genetic root of many cancers. While technical challenges remain for therapeutic application, proof-of-concept studies have demonstrated successful correction of clinically relevant mutations in cellular models [148]. If successfully translated, prime editing could enable true genetic reversion, restoration of normal genotype and phenotype, though delivery challenges and off-target effects must be overcome.

Beyond editing the genetic code, CRISPR-based epigenome editing enables targeted modification of gene expression without altering DNA sequence, directly relevant to reversion strategies that aim to restore normal epigenetic states. Konermann et al. [149] developed CRISPR activation (CRISPRa) systems using catalytically dead Cas9 (dCas9) fused to transcriptional activators, enabling precise activation of gene expression. Similarly, Qi et al. [150] developed CRISPR interference (CRISPRi), where dCas9 is fused to transcriptional repressors to silence target genes. This approach is particularly relevant for cancer reversion, as many cancers feature epigenetic dysregulation rather than genetic mutations as their primary driver. CRISPR-mediated epigenome editing has successfully reactivated silenced tumor suppressor genes and repressed overactive oncogenes in experimental models. For instance, targeted reactivation of the cell cycle regulator p21 using CRISPR activators induced cell cycle arrest and differentiation in various cancer cell lines [151]. The key question for reversion applications is durability: do CRISPR-mediated epigenetic changes persist after the editing machinery is removed? Current evidence suggests variable stability depending on the specific target and cellular context, with some epigenetic edits showing sustained effects and others requiring continuous presence of the editing machinery to maintain altered gene expression.

CRISPR technology also facilitates creation of sophisticated cellular and animal models that recapitulate the genetic complexity of human cancers, enabling detailed study of cancer development and testing of reversion strategies. CRISPR-engineered organoids provide physiologically relevant platforms for investigating cancer cell plasticity and responses to differentiating agents [152,153]. As delivery methods improve, including viral vectors, lipid nanoparticles, and cell-penetrating peptides, therapeutic application of CRISPR for cancer reversion moves closer to clinical reality.

### 5.3. Organoid Models

Patient-derived organoids have emerged as sophisticated 3D culture systems that bridge the gap between traditional cell lines and animal models. By recapitulating key aspects of tissue architecture and cellular heterogeneity, organoids provide unprecedented opportunities for studying cancer biology and testing reversion strategies in physiologically relevant contexts. Importantly, organoids enable assessment of whether reversion strategies produce durable phenotypic changes in complex, tissue-like environments that better mimic in vivo conditions.

Olawade et al. [152] extensively reviewed cancer organoid literature, reporting that patient-derived cancer organoids preserve the genetic and phenotypic heterogeneity of primary tumors to a remarkable degree. Unlike conventional cell lines, which represent highly selected subpopulations adapted to artificial culture conditions, organoids maintain the cellular diversity present in original tumors. This feature proves particularly valuable for cancer reversion research, as it enables study of how different cell populations within a tumor respond to reversion-inducing interventions. Colorectal cancer organoids have been shown to recapitulate the differentiation hierarchies found in primary tumors, with distinct subpopulations exhibiting varying degrees of stemness and differentiation [154], providing an ideal platform for testing differentiation therapies.

The predictive power of organoid models for clinical responses represents another significant advantage. Vlachogiannis and colleagues [155] demonstrated remarkable concordance between drug responses in gastrointestinal cancer organoids and clinical outcomes in the corresponding patients. Their “living biobank” of patient-derived organoids predicted with high accuracy which patients would benefit from specific treatments, suggesting similar applications for reversion-based therapies. For reversion strategies, organoids offer unique advantages: (1) Long-term culture capability allows assessment of phenotypic stability, do treated organoids maintain differentiated phenotypes for weeks to months in culture? (2) Serial passaging tests self-renewal capacity, do treated organoids lose tumor-initiating potential? (3) Transplantation into immunocompromised mice assesses tumorigenicity after treatment withdrawal. These approaches distinguish stable reversion from transient plasticity in a patient-specific manner.

Advanced co-culture systems incorporating multiple cellular components have further enhanced organoid model relevance. Neal and colleagues [156] developed air-liquid interface organoids that support co-culture of epithelial cancer cells with stromal and immune components, enabling study of complex cellular interactions that influence cancer behavior. These models can recapitulate features of the tumor microenvironment known to impact cancer cell differentiation and phenotypic plasticity. Pancreatic cancer organoids co-cultured with cancer-associated fibroblasts exhibit enhanced resistance to conventional therapies but may present unique vulnerabilities to reversion approaches targeting tumor-stroma interactions [157].

Technological innovations continue to enhance organoid utility for cancer reversion research: microfluidic systems enable precise control of the organoid microenvironment; bioprinting approaches facilitate creation of complex tissue architectures [152]; integration with CRISPR allows creation of isogenic organoid lines differing only in specific genetic elements [153], enabling precise determination of how genetic factors influence susceptibility to reversion strategies.

### 5.4. Artificial Intelligence and Machine Learning

The explosion of high-dimensional biological data has created both opportunities and challenges for cancer reversion research. AI and machine learning approaches offer powerful tools for extracting meaningful patterns from complex datasets, identifying novel therapeutic targets, and predicting cellular responses to potential reversion agents. Critically, AI can identify molecular signatures that distinguish stable reversion from transient plasticity, enabling rational design of interventions that produce durable phenotypic normalization.

Deep learning algorithms have demonstrated remarkable success in identifying compounds that induce differentiation in cancer cells. Chen and colleagues [158] developed a systems-based computational framework that integrated gene expression data from cancer samples and drug treatments to identify small molecules capable of reverting cancer-specific gene expression signatures toward normal patterns. Their approach predicted four compounds with high potency to reverse gene expression in liver cancer, and all four were validated as effective in five liver cancer cell lines. Similar approaches have been applied to repurpose existing drugs for cancer reversion, leveraging vast repositories of transcriptional response data [159,160]. Machine learning models can now predict not only which compounds induce phenotypic changes, but also which changes are likely to be stable vs. reversible based on patterns in gene expression, epigenetic, and signaling data.

Network analysis represents another powerful computational approach for dissecting regulatory systems that sustain malignant phenotypes. Cangiano [161] applied network inference methods to prostate cancer, identifying master regulators orchestrating transitions between cellular states. By integrating genomic, epigenomic, transcriptomic, and proteomic information, these models capture the multilayered regulatory architecture underpinning cellular plasticity, critical for reversion strategies since malignant states emerge from coordinated activity across multiple regulatory modules.

Building upon these insights, Gong and colleagues [60] advanced the concept of “network controllability” in cancer by employing computational models to identify reversion trajectories in colorectal cancer. Using single-cell transcriptomic data and network control theory, they mapped the dynamic landscape of cellular states and demonstrated that targeted perturbation of specific nodes could redirect malignant cells toward normal-like states. Their framework moves beyond static network descriptions to incorporate dynamic features, offering a blueprint for rationally designing interventions that restore homeostatic cell states.

Predictive modeling further underscores translational potential. Wang and colleagues [162] developed a deep neural network integrating multi-omics datasets (gene expression, copy number, mutations, proteomics, metabolomics) for predicting cellular responses to anticancer drugs in preclinical models, achieving R^2^ = 0.90. Such algorithms could identify patients most likely to benefit from reversion-based therapies and inform rational design of combination strategies tailored to individual molecular contexts.

AI-guided screening can also accelerate discovery iteratively: AI identifies candidate reversion agents, experimental validation generates data, refines AI models, identifies next-generation candidates. Advanced algorithms can detect subtle changes in cellular organization and tissue architecture signaling phenotypic normalization [163,164], including features predictive of stability such as terminal differentiation morphology, loss of invasive protrusions, and restoration of organized tissue architecture.

## 6. Challenges and Limitations

The concept of cancer reversion therapy, despite its considerable promise, faces substantial hurdles that must be addressed before it can achieve widespread clinical implementation. These challenges span from fundamental biological complexities to practical therapeutic considerations. Understanding these limitations is essential for realistic assessment of the field’s current state and for prioritizing research efforts toward overcoming key obstacles.

### 6.1. Cancer Cell Plasticity and Adaptive Resistance

Cancer cell plasticity represents perhaps the most significant biological challenge to reversion therapy. Cancer cells possess remarkable adaptability, allowing them to transition between different phenotypic states in response to therapeutic pressures. This inherent plasticity enables cancer cells to evade reversion attempts through compensatory mechanisms and adaptive responses. For example, differentiation-promoting therapies can initially induce phenotypic normalization in breast cancer, yet tumors often escape this through activation of alternative signaling pathways that preserve cancer stem cell traits such as stemness and malignancy. Canonical pathways including Wnt/β-catenin, Notch, Hedgehog, PI3K/Akt/mTOR, TGF-β, and NF-κB are well-established mediators of CSC self-renewal, survival, and drug resistance [165,166,167,168,169]. Similarly, in glioblastoma, differentiation-inducing therapies can fail over time due to epigenetic adaptations. Stress-induced chromatin remodeling (e.g., via EZH2, H3K27ac modifications) and downstream transcriptional shifts (e.g., STAT3 activation) promote return to a resilient glioma stem cell phenotype [170].

This plasticity fundamentally challenges the stability of reversion: even when therapeutic interventions successfully induce differentiation or phenotypic normalization, cancer cells may retain the capacity to dedifferentiate or transdifferentiate into resistant states. Therefore, understanding and effectively targeting the molecular underpinnings of this adaptability, including epigenetic modifications, transcriptional networks, and microenvironmental influences, is crucial for designing durable reversion therapies that prevent reemergence of malignancy.

### 6.2. Establishing and Validating Stable Reversion States

The identification and characterization of stable reversion states pose fundamental challenges. For reversion therapy to achieve lasting clinical benefit, cancer cells must transition to states that remain stable even after withdrawal of the therapeutic intervention. However, distinguishing truly stable reversion from temporary suppression of malignant features has proven difficult. Felsher [124] articulated this challenge through the concept of “oncogene addiction versus amnesia,” noting that some cancers rapidly revert to aggressive behavior once treatment pressure is removed.

The stability of reverted states likely depends on complex factors including:

- Genetic background: Cells with fewer oncogenic mutations may be more amenable to stable reversion;
- Epigenetic landscape: Stable DNA methylation changes predict durable reversion; transient histone modifications suggest reversibility;
- Microenvironmental context: Supportive stromal signals may maintain normalized phenotypes; permissive environments may allow relapse;
- Treatment history: Duration and intensity of reversion-inducing therapy may determine epigenetic consolidation;
- Differentiation stage: Terminal differentiation (e.g., ATRA in APL) produces irreversible reversion; partial differentiation may be reversible.

Experimental approaches using lineage tracing combined with single-cell analyses now offer promising methods for tracking cellular trajectories and identifying conditions that promote stable transitions [137]. However, translating these insights into clinical strategies remains challenging, particularly given the extended timeframes needed to assess stability in human patients. Current gaps include:

- Lack of validated biomarkers predicting stability vs. reversibility before treatment withdrawal;
- Insufficient long-term follow-up data in most experimental and clinical studies;
- Need for standardized criteria defining “stable reversion” with minimum follow-up durations;
- Limited understanding of molecular mechanisms that lock in vs. allow escape from differentiated states.

### 6.3. Delivery Challenges for Solid Tumors

Technical challenges related to therapeutic delivery present significant obstacles, particularly for solid tumors. Many promising reversion-inducing agents, including biologics, nucleic acids, and epigenetic modifiers, exhibit poor pharmacokinetics, limited tissue penetration, and unfavorable toxicity profiles. Dense ECM, abnormal vasculature, and high interstitial pressure impede delivery of therapeutic agents [171]. Even when agents reach tumor cells, achieving sustained exposure necessary for cellular reprogramming without causing systemic toxicity presents major challenges.

Innovative delivery approaches show promise: nanoparticle formulations, antibody-drug conjugates, and localized delivery devices. Bertrand et al. [172] described ring-opening metathesis polymerization (ROMP) as a powerful strategy to synthesize pH-sensitive nanoparticles for tumor therapy, enabling controlled, targeted drug release in acidic tumor microenvironments. However, significant optimization of delivery technologies will be required to translate laboratory findings into clinically effective reversion therapies, particularly for:

- Achieving therapeutic concentrations in poorly vascularized tumor regions;
- Maintaining sustained drug exposure over weeks to months required for epigenetic reprogramming;
- Minimizing off-target effects in normal tissues with high proliferation rates (bone marrow, GI tract);
- Crossing biological barriers (blood–brain barrier for CNS tumors).

### 6.4. Tumor Heterogeneity and Need for Combination Approaches

The profound heterogeneity of cancer, both between and within individual tumors, necessitates combination approaches that target multiple cellular subpopulations and signaling pathways simultaneously. Single-agent reversion therapies have typically shown limited efficacy in heterogeneous cancers. Koren and Bentires-Alj [173] highlighted how intratumoral heterogeneity enables resistant subpopulations to expand under therapeutic pressure, leading to treatment failure.

Rational combination strategies are essential for effective reversion therapy, potentially including:

- Agents targeting different cellular subpopulations (e.g., differentiated vs. stem-like cancer cells);
- Simultaneous modulation of multiple regulatory networks (e.g., epigenetic + signaling pathway inhibition);
- Addressing both cancer cells and supportive microenvironment (e.g., differentiation therapy + macrophage reprogramming).

Principe et al. [174] demonstrated synergistic effects when combining epigenetic modifiers with microenvironment-targeting agents in pancreatic cancer models, achieving more complete and durable reversion than either approach alone. However, designing optimal combinations requires:

- Sophisticated understanding of interaction mechanisms (synergy vs. antagonism);
- Careful management of combined toxicities;
- Complex clinical development pathways and regulatory considerations;
- Determination of optimal sequencing and scheduling.

### 6.5. Clinical Trial Design and Endpoint Challenges

Developing reversion therapies faces unique clinical trial challenges. The optimal timing and sequencing of reversion therapy within broader treatment regimens remains poorly defined. Additionally, current response criteria in oncology trials predominantly focus on tumor shrinkage, which may inadequately capture benefits of therapies that normalize cell behavior without necessarily reducing tumor size.

Critical challenges include:

- Defining appropriate primary endpoints: Overall survival and progression-free survival may not capture early reversion responses; differentiation markers, MRD, functional recovery are candidate surrogate endpoints requiring validation.
- Extended observation periods: Assessing stability requires longer follow-up than typical phase II trials (minimum 6–12 months post-treatment cessation).
- Biomarker development: Robust, validated biomarkers distinguishing stable reversion from transient plasticity are needed.
- Patient selection: Identifying which patients are most likely to achieve stable reversion based on tumor biology, genetic/epigenetic profiles.
- Combination trial complexity: Testing multiple agent combinations with various schedules creates factorial complexity.
- Regulatory pathways: Gaining regulatory acceptance for novel endpoints beyond tumor regression.

Developing imaging approaches, molecular assays, and functional assessments that can reliably measure phenotypic normalization in clinical settings will be essential for advancing reversion therapies through clinical development.

### 6.6. Economic and Developmental Barriers

Economic considerations present challenges, as pharmaceutical development typically prioritizes treatments with conventional cytotoxic mechanisms demonstrating rapid tumor responses in clinical trials. Development of reversion therapies may require:

- Longer-term investment with delayed demonstration of benefit;
- Alternative approaches to demonstrating clinical benefit beyond rapid tumor shrinkage;
- More expensive biomarker assessments (epigenetic profiling, single-cell analyses);
- Extended clinical trial durations to assess stability.

These factors create barriers to commercial development despite scientific promise, potentially requiring:

- Public–private partnerships;
- Academic-led trials with alternative funding mechanisms;
- Regulatory incentives for novel therapeutic paradigms;
- Health economic modeling demonstrating long-term value (e.g., reduced toxicity, improved quality of life, potential for treatment-free remission).

Despite these substantial challenges, they represent research priorities rather than insurmountable barriers. Addressing these limitations will require interdisciplinary collaboration, innovative technologies, and creative clinical trial designs. The potential rewards, treatments that normalize cancer cells with reduced toxicity and resistance, justify continued pursuit of this promising therapeutic paradigm.

Table 3 contrasts conventional cancer trial designs with reversion therapy requirements, emphasizing that reversion trials demand novel endpoints (differentiation markers, stability metrics), extended post-treatment monitoring (6–24 months after cessation), biomarker-driven patient selection, and regulatory frameworks accepting phenotypic normalization rather than tumor shrinkage as meaningful clinical benefit.

Evidence Classification:

- (Established evidence) = Standard practice with regulatory acceptance and extensive clinical experience;
- (Emerging/moderate evidence) = Some clinical precedent, published data, or early regulatory examples exist;
- (Speculative) = Theoretical considerations or proposed approaches lacking substantial validation.

Key Considerations for Trial Implementation:

1. Stability assessment is paramount: Trials must include planned treatment withdrawal phases with extended post-cessation monitoring;
2. Biomarker development is critical: Validated assays distinguishing stable reversion from transient plasticity are urgently needed;
3. Patient selection refinement: Predictive biomarkers identifying patients likely to achieve stable vs. transient responses will improve trial efficiency;
4. Novel endpoint validation: Regulatory acceptance of reversion-specific endpoints requires robust correlation with clinical benefit;
5. Combination complexity: Rational sequencing and timing of combination regimens requires mechanistic understanding and pharmacodynamic monitoring.

## 7. Future Directions

Table 4 explore combination approaches that may enhance cancer reversion efficacy. Table 4—(A) presents strategies with existing preclinical or early clinical evidence, while Table 4—(B) explores conceptual proposals requiring experimental validation. These combinations address the reality that single-agent reversion approaches often produce incomplete or reversible effects, while rational combinations may achieve more stable phenotypic normalization.

Interpretation:

- Table 4—(A) strategies have evidence supporting mechanistic rationale and feasibility; represent near-term translational opportunities.
- Table 4—(B) strategies are conceptual proposals with plausible biological rationale but lacking experimental validation; represent longer-term research directions.
- Common theme: Combinations address fundamental limitation that single agents often produce incomplete or reversible reversion; rational combinations may achieve stable phenotypic normalization by simultaneously targeting multiple maintenance mechanisms.
- Stability enhancement rationale is critical: combinations should not merely add efficacy but specifically address mechanisms that allow dedifferentiation or phenotypic reversion.

### 7.1. Integrated Multi-Omics Approaches

The complexity of cancer reversion demands comprehensive analytical approaches capturing the multidimensional nature of cellular state transitions. Integrated multi-omics strategies combining different molecular data types offer unprecedented insights into mechanisms governing cancer cell plasticity and factors inducing reversion to non-malignant states. Critically, multi-omics integration can distinguish stable reversion from transient plasticity by revealing coordinated changes across genomic, epigenomic, transcriptomic, proteomic, and metabolomic layers characteristic of consolidated phenotypic transitions.

For example, in hepatocellular carcinoma, integrative analysis of genomic, transcriptomic, proteomic, and metabolomic data across cell lines with varying metastatic potential identified dysregulated metabolic pathways, including consistently elevated UGP2, representing a metabolic dependency that could be targeted to reduce malignancy [193]. Similarly, in colorectal cancer, proteogenomic profiling of paired primary tumors and liver metastases revealed proteomic divergence and signaling pathway alterations guiding differential drug responses in xenograft models [194], suggesting that integrative profiling identifies stage-specific vulnerabilities for therapeutic intervention or reversion. An integrative study of colon cancer invasiveness identified transcriptional reprogramming events mediated by transcription factors like JunD that drive metastatic behavior [195]. These regulators of chromatin state and gene expression may serve as actionable nodes for reversion therapy, whereby restoring or redirecting transcriptional programs could return cells to less aggressive phenotypes.

Multi-omics trajectory analysis represents another powerful approach for understanding cancer reversion. These computational methods infer developmental trajectories from single-cell multi-omics data, enabling detailed mapping of cellular state transitions [196]. In hematological malignancies, these approaches have elucidated molecular changes as cells progress from normal to pre-malignant to fully malignant states, identifying potential intervention points where progression might be reversed. Single-cell sequencing revealed that clonal complexity increases as myeloid malignancies progress, with specific co-occurring mutations associated with clonal dominance [197]. Analyses have shown that certain transcription factors act as “decision points” in cellular trajectories, where targeted intervention could potentially redirect cells toward normal differentiation paths. For example, in TP53-mutant secondary AML, single-cell transcriptomics revealed distinct differentiation trajectories and molecular features [198]. Similar approaches in solid tumors have identified state transitions associated with therapy resistance and metastasis. Single-cell multi-omics identified metabolism-linked epigenetic reprogramming as a driver of therapy-resistant medulloblastoma [199]. In gastric cancer, single-cell transcriptomics revealed multidimensional dynamic heterogeneity from primary to metastatic stages, identifying key transcription factors facilitating tumor cell migration [200].

Systems biology approaches further enhance understanding by identifying key regulatory nodes controlling cellular states. Network-based methods identify master regulator proteins orchestrating broad transcriptional programs determining cellular phenotypes. Analyses across cancer types reveal that although genetic alterations are diverse, they often converge on smaller sets of regulatory modules maintaining malignant states [201]. Targeting these master regulators, rather than individual genetic alterations, may provide more effective strategies for inducing cancer reversion. Identification of FOXM1 and CENPF as synergistic master regulators in aggressive prostate cancer showed that co-targeting these factors could revert malignant phenotypes despite underlying genetic alterations [202].

Future evolution of integrated multi-omics will likely incorporate spatial information, temporal dynamics, and functional readouts alongside molecular data, revealing how location within tumors influences cellular states and reversion potential. Longitudinal sampling during treatment captures dynamic processes of resistance and adaptation, identifying optimal timing for reversion interventions. Integration of functional assays with molecular profiles enhances predictive models of cellular behavior under therapeutic pressure.

### 7.2. Leveraging Natural Examples of Reversion

Nature provides remarkable examples of cancer reversion offering valuable lessons for therapeutic development. Rare spontaneous regression cases, developmental contexts suppressing malignancy, and physiological states inducing cancer normalization represent natural experiments informing rational design of reversion therapies.

Spontaneous regression of melanoma offers particularly instructive examples. Immune profiling of melanoma lesions undergoing spontaneous regression revealed distinctive immune signatures characterized by coordinated activity of specific T cell subsets, natural killer cells, and antigen-presenting cells [203,204,205]. These analyses identified not only cytotoxic mechanisms but also immune-mediated differentiation pathways appearing to reprogram rather than simply eliminate cancer cells. The nCounter technology has been used to evaluate gene signatures in melanoma, with a 53-gene immune signature predicting non-progression and prolonged survival [206]. Recent advances in spatially resolved immune profiling now enable more precise characterization of cellular and molecular interactions underlying these phenomena [207,208], potentially revealing new therapeutic targets for inducing reversion through immune modulation.

Neuroblastoma, a pediatric cancer with unusually high spontaneous regression rates, provides another valuable model. Brodeur and Bagatell [209] reviewed extensive evidence regarding biological basis of neuroblastoma regression, highlighting roles of neurotrophic factors, developmental timing, and sympathetic nervous system maturation. Their work suggested that specific microenvironmental factors present during normal sympathetic nervous system development can override oncogenic signals in neuroblastoma cells, inducing differentiation or apoptosis, representing true stable reversion in natural settings. These insights led to experimental therapies using neurotrophin analogs and retinoic acid derivatives to recapitulate natural regression. Ongoing single-cell analyses of spontaneously regressing neuroblastomas promise to further elucidate precise molecular mechanisms, potentially revealing principles applicable to other cancer types.

Beyond specific examples, comparative studies across multiple types of naturally occurring reversion could reveal common principles. Comprehensive molecular characterization of diverse regression phenomena, including radiation-induced abscopal effects, post-infectious tumor regressions, and tissue-specific differences in cancer progression, may identify shared pathways mediating reversion across different contexts. Such analyses could distinguish fundamental reversion mechanisms from context-specific factors, guiding development of broadly applicable therapeutic strategies.

### 7.3. Nanotechnology-Based Delivery Systems

Effective delivery of reversion-inducing agents represents a critical challenge, particularly for solid tumors. Advanced nanotechnology platforms offer promising solutions by enhancing specific targeting, improving pharmacokinetics, and enabling controlled release of therapeutic agents at tumor sites. Importantly, successful reversion often requires sustained drug exposure over weeks to months to achieve epigenetic consolidation; nanoparticle systems enabling controlled release are ideally suited for this application.

Nanoparticle-based delivery of epigenetic modifiers has shown promise for cancer reversion applications. El Bahhaj and colleagues [210] developed polymeric micelle hybrid nanoparticles encapsulating HDAC inhibitors demonstrating significantly improved tumor accumulation and reduced systemic toxicity compared to free drug. These nanoformulations not only enhanced anti-tumor efficacy but also increased expression of differentiation markers in cancer cells, suggesting improved reversion activity. The ability of nanoparticles to protect sensitive compounds from degradation while enabling sustained release at tumor sites makes them particularly valuable for epigenetic reprogramming approaches, which typically require extended exposure to induce stable phenotypic changes. Similar approaches have been applied to other epigenetic modulators with promising results across multiple cancer models.

Biomimetic nanoparticles represent an innovative approach for targeting specific tumor microenvironments. Nanoparticles coated with cancer cell membranes enable homotypic targeting through cell–cell recognition mechanisms [211]. These particles demonstrate remarkable specificity for their cells of origin, suggesting applications for delivering reversion agents to particular cancer cell populations within heterogeneous tumors. Other biomimetic approaches include nanoparticles coated with ECM components, platelet-mimetic particles targeting circulating tumor cells, and leukocyte-mimetic particles penetrating inflammatory tumor microenvironments [211]. These sophisticated targeting strategies could enable precise delivery of reversion agents to specific cellular subpopulations or microenvironmental niches maintaining the malignant state.

Stimuli-responsive delivery systems offer another advanced approach for targeting reversion therapies. Majumder and Minko [212] reviewed various stimuli-responsive nanoparticles designed to release therapeutic cargo in response to specific tumor microenvironment features such as acidic pH, elevated matrix metalloproteinases, or hypoxia. These systems enable site-specific activation of reversion agents, minimizing off-target effects while achieving higher local concentrations at tumor sites. For example, pH-sensitive nanoparticles can shield reversion-inducing agents during circulation but release them upon encountering acidic tumor microenvironments. These approaches allow precise spatiotemporal control over drug release, potentially enhancing efficacy and safety of reversion therapies.

Integration of diagnostic and therapeutic capabilities within single nanotechnology platforms, “theranostic” approaches, holds promise for cancer reversion strategies. Nanoparticles incorporating imaging agents alongside therapeutic compounds enable real-time monitoring of drug delivery and early assessment of cellular responses to reversion therapy. These capabilities prove especially valuable for reversion approaches, where traditional response criteria based on tumor shrinkage may inadequately capture therapeutic effects. Molecular imaging of differentiation markers, metabolic changes, or altered cellular organization could provide early indicators of successful reversion, guiding treatment decisions and dosing schedules.

### 7.4. Synthetic Biology Approaches

Synthetic biology offers revolutionary approaches to cancer reversion by engineering biological systems with novel functionalities designed specifically to detect cancer cells and trigger reversion programs. These approaches harness cellular machinery itself as both detection system and therapeutic effector.

Synthetic gene circuits represent a sophisticated approach for cancer-specific reversion therapy. Newly designed genetic circuits can identify cancer cells based on molecular signatures and activate customized response programs. Systems utilize multiple inputs, including microRNA levels, transcription factor activities, and signaling pathway states, to distinguish cancer cells from normal tissues with high specificity [213,214]. Upon detecting cancer-specific signatures, these circuits activate tailored response programs inducing differentiation, apoptosis, or immunogenic cell death depending on cellular context. In glioblastoma models, circuits detecting Sox2 and Oct4 activity triggered expression of neural differentiation factors, effectively reprogramming cancer stem cells toward less aggressive phenotypes [215]. Similar approaches could be designed for various cancer types, with circuit components optimized to detect specific molecular features and trigger appropriate reversion programs, potentially providing highly specific, self-regulating reversion therapies.

Cell-based therapies leveraging synthetic biology principles offer another promising direction. Roybal and colleagues [216] developed engineered T cells with synthetic receptors and customized response programs recognizing specific cancer markers and executing sophisticated functions beyond simple cytotoxicity. While initially focused on traditional immunotherapy, these systems could be adapted to deliver reversion-inducing factors to cancer cells. For instance, engineered cells could recognize cancer-specific antigens and secrete differentiation-inducing cytokines, matrix-modifying enzymes, or exosomes containing epigenetic regulators. The ability of cells to actively migrate through tissues, persist for extended periods, and respond dynamically to changing conditions offers advantages over conventional drug delivery. Recent advances enable creation of “living therapeutics” with multiple sensing and response capabilities, potentially allowing intelligent delivery of reversion factors adapted to specific needs of individual tumors.

Targeted protein degradation represents another synthetic biology approach with significant implications for cancer reversion. Bondeson and colleagues [217] developed PROTACs, bifunctional molecules that tag specific proteins for degradation by the ubiquitin-proteasome system. This technology enables selective removal of oncogenic drivers and epigenetic regulators maintaining malignant state, potentially allowing restoration of normal cellular programming. Unlike conventional inhibitors requiring continuous presence to suppress protein function, PROTACs achieve sustained effects through actual elimination of target proteins, particularly valuable for cancer reversion, where transient disruption of key regulatory nodes might initiate self-sustaining differentiation programs. Recent advances in PROTAC design have expanded the range of targetable proteins and improved cellular penetration and stability.

Integration of synthetic biology with other advanced technologies promises even more sophisticated reversion strategies: combinations of engineered cellular systems with responsive biomaterials could create artificial niches promoting cancer cell differentiation; synthetic gene circuits coupled with optogenetic or chemogenetic control systems could enable precise spatiotemporal regulation of reversion factors; cell-free synthetic biology approaches, including engineered extracellular vesicles carrying therapeutic cargoes, offer alternatives to whole-cell therapies with potentially favorable manufacturing and regulatory profiles [218]. As synthetic biology tools become more sophisticated and our understanding of cancer cell states deepens, increasingly precise and effective reversion strategies will likely emerge from this rapidly evolving field.

The future of cancer reversion therapy lies at the intersection of these four directions, integrated multi-omics, natural reversion examples, advanced delivery systems, and synthetic biology. Their convergence creates unprecedented opportunities to understand, target, and reprogram the cancer cell state with precision and sophistication. While significant challenges remain, the accelerating pace of innovation across these domains suggests that cancer reversion therapy may yet fulfill its promise as a transformative approach to cancer treatment.

## 8. Limitations of the Review

This narrative review on cancer reversion therapy, while comprehensive in scope, has several important limitations that should be acknowledged:

1. Rapid Field Evolution and Publication Bias: the rapidly evolving nature of cancer biology and therapeutic development means that new findings continue to emerge at an accelerating pace. Despite our thorough literature search (January 1997–August 2025), some recent discoveries may not have been incorporated. Publication bias likely influences our analysis: studies demonstrating successful cancer reversion are more likely to be published than those showing negative or inconclusive results, potentially creating an overly optimistic portrayal of the field’s progress. Without access to unpublished data or comprehensive clinical trial results (including discontinued trials), our assessment may not fully represent the actual state of the field.

2. Conceptual Boundaries and Terminology Inconsistency: the conceptual boundaries of what constitutes “cancer reversion” remain somewhat fluid in scientific literature. We have attempted to address this by establishing explicit definitions distinguishing stable reversion from stimulus-dependent plasticity, dormancy, and cytotoxic responses (Section 1.1). However, many published studies predate this framework and use “reversion” terminology inconsistently. We applied subjective judgment in classifying studies, potentially influencing conclusions about current status and prospects. Our framework represents one interpretation; alternative classification schemes might yield different assessments of which interventions produce true stable reversion.

3. Limited Clinical Outcome Data: our review primarily focused on mechanistic studies and technological developments rather than systematic assessment of clinical outcomes. The relative scarcity of completed clinical trials specifically designed to assess cancer reversion endpoints (especially long-term stability after treatment withdrawal) limits our ability to draw firm conclusions about therapeutic efficacy. Many agents with potential reversion activity have been evaluated clinically using conventional endpoints (tumor regression, PFS) which may not adequately capture phenotypic normalization effects. This creates uncertainty: interventions showing differentiation-inducing activity in preclinical models may or may not produce clinically meaningful, durable reversion in patients.

4. Heterogeneity in Experimental Approaches: studies range from 2D cell cultures to complex animal models to limited human data, with varying methodologies for assessing reversion phenotypes. Most critically, many studies lack adequate post-treatment follow-up to definitively classify responses as stable reversion vs. transient plasticity. This heterogeneity creates challenges for synthesizing findings into coherent mechanistic models or therapeutic recommendations applicable across cancer types. Our evidence classification system (Level 1–3, Section 2.4) represents an attempt to address this, but assignment of evidence levels involves subjective interpretation.

5. Geographical and Language Limitations: our focus on English-language publications may have excluded relevant findings from non-English sources, potentially limiting geographical and cultural diversity in research perspectives. Cancer biology and treatment approaches can vary across populations, and important insights from diverse research communities may have been overlooked.

6. Narrative Review Methodology: as a narrative rather than systematic review, our synthesis inherently involves subjective interpretation and emphasis. While we endeavored to provide balanced coverage, author expertise and interests may have influenced relative attention given to different topics or interpretation of conflicting evidence. Unlike systematic reviews with pre-specified protocols, narrative reviews are susceptible to selection and reporting biases.

7. Insufficient Stability Data Across Literature: perhaps most critically for assessing cancer reversion, we found that the majority of published studies lack adequate assessment of phenotypic stability after treatment withdrawal. Most studies report observations during treatment exposure or immediately upon treatment cessation, with limited long-term follow-up. This makes it difficult to definitively classify many reported “reversion” phenomena as stable vs. transient across the literature. Our classifications (Table 1, throughout manuscript) represent best assessments based on available data, but many require further validation with extended post-treatment observation periods.

These limitations highlight the need for:

- Standardized terminology and assessment criteria for reversion phenotypes;
- Minimal standards for post-treatment follow-up duration in experimental and clinical studies;
- Systematic reviews and meta-analyses where appropriate;
- Comprehensive clinical trial reporting including negative results;
- International research collaboration;
- Integration of findings across diverse experimental systems and cancer types.

Future reviews would benefit from these improvements, enabling more definitive assessments of which therapeutic strategies produce true stable reversion vs. alternative phenomena.

## 9. Conclusions

Cancer reversion therapy represents a promising frontier in oncology, offering the potential for therapeutic approaches characterized by reduced toxicity and diminished likelihood of resistance compared to conventional treatments. However, realizing this potential requires careful distinction between interventions producing truly stable, durable phenotypic normalization and those inducing transient, reversible changes requiring continuous therapeutic pressure.

The current state of the field reveals a spectrum of reversion outcomes:

- Definitive successes: ATRA in APL demonstrates that terminal differentiation producing stable, durable remissions is achievable, providing proof-of-principle for cancer reversion as a therapeutic paradigm.
- Partial successes: BCR-ABL inhibition in CML shows that 40–60% of patients can achieve treatment-free remission, while others require continuous therapy, highlighting that stable reversion is context-dependent.
- Promising but unvalidated approaches: Many epigenetic, microenvironmental, and differentiation-based strategies show encouraging preclinical results, but most lack adequate long-term data confirming stable reversion in patients.
- Stimulus-dependent plasticity: Numerous interventions (ECM normalization, vascular normalization, macrophage reprogramming, and some differentiation agents) produce phenotypic normalization only while treatment is maintained, representing valuable combination therapy components but not standalone reversion strategies.

Significant scientific and clinical challenges remain:

- Cancer cell plasticity enables escape from partially induced differentiation states;
- Distinguishing stable reversion from transient plasticity requires extended post-treatment observation periods rarely performed in current studies;
- Delivery challenges limit solid tumor applications;
- Tumor heterogeneity necessitates combination approaches;
- Clinical trial designs and regulatory pathways need adaptation for reversion-specific endpoints.

However, recent advances create unprecedented opportunities to develop viable cancer reversion strategies:

- Single-cell analyses can map cellular state transitions and identify stable vs. transient phenotypic changes;
- CRISPR-based approaches enable precise genetic and epigenetic manipulation to test reversion mechanisms;
- Organoid models recapitulate tumor complexity and enable assessment of phenotypic stability;
- AI/machine learning can predict which interventions produce durable reversion;
- Nanotechnology enables sustained delivery of reversion-inducing agents required for epigenetic consolidation;
- Synthetic biology approaches offer intelligent, self-regulating therapeutic systems.

Moving forward, achieving the transformative vision of cancer reversion as a clinical reality depends critically on the following:

- Establishing rigorous criteria and standardized assessment methods for stable reversion (extending beyond the treatment period);
- Developing and validating biomarkers distinguishing stable reversion from transient plasticity (especially epigenetic stability markers);
- Rational combination strategies that consolidate phenotypic changes induced by individual agents;
- Extended follow-up in experimental and clinical studies (minimum 3–6 months post-treatment in preclinical; 12–24 months in clinical);
- Interdisciplinary collaboration among basic researchers, clinicians, bioengineers, and technology developers;
- Innovative clinical trial designs with reversion-appropriate endpoints and observation periods;
- Regulatory pathways accepting novel endpoints beyond tumor shrinkage.

The field stands at a critical juncture: the biological plausibility of cancer reversion is established, proof-of-principle exists (APL, subset of CML patients), technological capabilities are expanding rapidly, but translating these advances into broadly applicable, clinically effective reversion therapies requires addressing fundamental questions about phenotypic stability and developing interventions that produce durable, not merely transient, normalization.

The ultimate promise, transforming cancer from a terminal diagnosis to a chronic, manageable condition through cellular reprogramming rather than destruction, justifies continued vigorous pursuit of this therapeutic paradigm. Success will require a realistic assessment of current limitations, rigorous experimental standards (especially regarding stability assessment), and sustained commitment to developing combination strategies that achieve true stable reversion. Continued expansion of our understanding of cancer’s molecular and cellular mechanisms, combined with emerging technologies, is making the concept of reprogramming malignant cells increasingly attainable, potentially revolutionizing cancer treatment for patients globally.

## Figures and Tables

**Table 1:** (A) Comparison of Cancer Reversion Strategies across Major Cancer Types—Hematological Malignancies; (B) Comparison of Cancer Reversion Strategies across Major Cancer Types—Solid Tumors.

(**A**)					
**Cancer Type**	**Predominant Strategy**	**Key Molecular Targets**	**Development Status**	**Notable Response Markers**	**Evidence of Stability**
Acute Myeloid Leukemia [54,55]	Differentiation therapy via LSD1 inhibition (±combination with epigenetic modulators)	LSD1 (KDM1A), GSK3/WNT pathway, RARα	Preclinical; early phase clinical trials	CD11b, CD86 myeloid markers; morphological maturation; reactivation of retinoic acid pathway genes	Level 2: 4–8 week stability in vitro; clinical durability under investigation
Acute Promyelocytic Leukemia [29,30,56,57]	ATRA + arsenic trioxide differentiation therapy	PML-RARα fusion protein, differentiation pathway genes	FDA-approved; standard of care	Terminal neutrophil differentiation; molecular remission; >95% complete remission rate	Level 1: Durable remissions >10 years; true stable reversion with terminal differentiation
Chronic Myeloid Leukemia [53,58,59]	BCR-ABL inhibition (imatinib, second-generation TKIs)	BCR-ABL tyrosine kinase, downstream signaling pathways	FDA-approved; standard of care	Complete cytogenetic remission; restoration of normal hematopoiesis	Level 2: 40–60% maintain remission off-therapy (“treatment-free remission”); subset shows stable reversion
(**B**)					
Colorectal Cancer [60,61,62]	Master regulator knockdown; epigenetic reprogramming via DNMT inhibition + statins	MYB, HDAC2, FOXA2, DNMTs, BMP2 promoter, Wnt/β-catenin	Preclinical (in vitro + xenograft)	Reduced proliferation; enterocyte-like gene expression; decreased stem cell markers; BMP2 reactivation	Level 3: Requires continuous treatment in most models; reversibility upon treatment withdrawal reported
Breast Cancer (General) [63,64,65]	Microenvironment-mediated MErT; ECM/integrin manipulation	E-cadherin (CDH1), vimentin, αvβ3-integrin, ECM components	Preclinical; experimental models	Re-expression of E-cadherin; morphological reversion; altered motility; demethylation of CDH1 promoter	Level 3: Phenotype maintained only in 3D/ECM context; rapid reversion (7–14 days) upon return to 2D culture
Breast Cancer (TNBC) [66,67]	HDAC inhibitors + natural compounds; COX-2/GSK3β pathway targeting	E-cadherin, Slug/Twist/Vimentin, COX-2, GSK3β, p120-catenin	Preclinical (in vitro, PDX models)	Increased epithelial markers; decreased mesenchymal markers; stabilized adherens junctions; reduced CTC clusters	Level 2–3: Some stability (2–4 weeks) in vitro; durability in vivo requires continuous treatment in most studies
Hepatocellular Carcinoma [68]	DNMT1 inhibition; low-dose demethylating agents (5-AZA)	DNMT1; hypermethylated hepatocyte-specific genes	Preclinical (HCC cell lines)	Increased hepatocyte-specific gene expression; restoration of hepatic functions; reduced malignancy markers	Level 3: Limited post-treatment follow-up data; stability beyond treatment period unclear
Melanoma [69]	MITF overexpression; transcriptional modulation	MITF, tyrosinase, TRP-1, proliferation/migration factors	Preclinical (in vitro, in vivo)	Melanocytic differentiation markers (tyrosinase, TRP-1); reduced proliferation (Ki-67↓); decreased metastasis in mouse models	Level 2: Phenotype maintained 3–4 weeks post-treatment in some models; subset shows reversibility
Basal Cell Carcinoma [70,71]	Hedgehog pathway inhibition (SMO inhibitors)	PTCH1, SMO, GLI1 (Hedgehog pathway)	FDA-approved (systemic); Phase II trials (topical)	Reduction in new BCC lesions; tumor size reduction; downregulation of GLI1, PTCH1; decreased HH signaling	Level 2: Clinical benefit requires continuous treatment; relapse common after cessation (stimulus-dependent)
Neuroblastoma [72]	TrkA overexpression/activation; neurotrophic signaling modulation	TrkA, TrkB, NGF, ATRA, RET pathway	Preclinical (cell lines; xenograft)	Growth arrest; neurite outgrowth; neuronal differentiation markers; TrkA upregulation; decreased migration/invasion	Level 2: Some evidence of stable differentiation; subset shows spontaneous regression (natural stable reversion example)

Abbreviations: ATRA, all-trans retinoic acid; BCC, basal cell carcinoma; BMP2, bone morphogenetic protein 2; CDH1, cadherin 1 (E-cadherin); COX-2, cyclooxygenase-2; CTC, circulating tumor cell; DNMT, DNA methyltransferase; ECM, extracellular matrix; GSK3β, glycogen synthase kinase 3 beta; HCC, hepatocellular carcinoma; HDAC, histone deacetylase; LSD1, lysine-specific demethylase 1; MErT, mesenchymal to epithelial reverting transition; MITF, microphthalmia-associated transcription factor; NGF, nerve growth factor; PDX, patient-derived xenograft; PTCH1, patched 1; SMO, smoothened; TKI, tyrosine kinase inhibitor; TNBC, triple-negative breast cancer; TrkA/B, tropomyosin receptor kinase A/B; TRP-1, tyrosinase-related protein 1.

**Table 2:** Biomarkers for Monitoring Cancer Reversion and Distinguishing Stable from Transient Changes.

Biomarker Category	Representative Examples	Detection/Assay	Clinical Utility	Stability Assessment Capability	Key Limitations
Morphological Changes [126]	Cell size/shape, nuclear-to-cytoplasmic ratio, tissue architecture, differentiation morphology	Pathology, H&E staining, advanced microscopy (confocal, multiphoton)	Direct visualization of phenotypic maturation; long historical use; can detect terminal differentiation features (e.g., segmented neutrophils in APL)	Moderate: Can identify terminal vs. partial differentiation; requires serial biopsies to assess stability	Requires tissue sampling; subjective interpretation; inter-observer variability; snapshot at single timepoint
Differentiation Markers [127]	Lineage-specific transcription factors (C/EBPα, PU.1), surface markers (CD11b, CD14, E-cadherin), lineage-restricted proteins (tyrosinase, cytokeratins)	Immunohistochemistry, flow cytometry, Western blot, qPCR	Quantifies differentiation state; tracks lineage commitment; can be monitored serially in blood/bone marrow	Good: Serial measurements can detect sustained vs. transient expression; loss of stemness markers (Oct4, Sox2, Nanog) indicates commitment	Context-dependent expression; heterogeneous within tumors; requires repeated sampling; surface markers may not reflect functional maturation
Epigenetic Signatures [128,129]	DNA methylation (5-mC, 5-hmC), histone marks (H3K27ac, H3K4me3), chromatin accessibility (ATAC-seq peaks)	Bisulfite sequencing (WGBS, RRBS), CUT&Tag, ChIP-seq, ATAC-seq, cfDNA methylation analysis	Early molecular readout of reprogramming; DNA methylation changes predict stability (more stable than histone modifications); liquid biopsy-compatible (cfDNA)	Excellent: DNA methylation = stable, heritable; H3K27ac/H3K4me3 = transient, reversible; Chromatin accessibility changes = intermediate stability; Serial monitoring distinguishes consolidation vs. reversal	Requires specialized sequencing; bioinformatics complexity; tissue or high-quality cfDNA needed; cost; interpretation challenges distinguishing driver vs. passenger changes
Functional Restoration Assays [130,131]	Tumor-initiating capacity (serial transplantation), colony formation assays, invasion/migration assays, tissue-specific functions (phagocytosis for neutrophils, insulin secretion for beta cells)	Serial limiting dilution transplantation, soft agar assays, transwell/Matrigel invasion, functional biochemical assays	Gold Standard for stable reversion: Loss of tumor-initiation in serial transplants = true reversion; Restoration of physiological functions = functional normalization	Excellent: Directly measures reversibility: Can cancer cells re-initiate tumors after treatment withdrawal? Can they perform normal cell functions?	Labor-intensive; requires animal models or long-term culture; expensive; not feasible for routine clinical monitoring; typically research use only
Metabolic Profiles [132,133]	Glycolysis (FDG uptake, lactate), oxidative phosphorylation (oxygen consumption), lipid metabolism (fatty acid oxidation), metabolomics signatures	FDG-PET, Seahorse assays, mass spectrometry-based metabolomics, novel PET tracers (e.g., glutamine, acetate)	Functional assessment of cellular state (differentiated cells show reduced glycolysis, increased OXPHOS); non-invasive imaging possible (FDG-PET)	Moderate: Sustained metabolic changes suggest phenotypic consolidation; reversible shifts indicate plasticity	Metabolic plasticity even in stable phenotypes; confounders (inflammation, necrosis); technical complexity; expensive; interpretation challenges
Spatial Organization/Architecture [134]	Tissue polarity (apical-basal markers), cell–cell junctions (adherens, tight, gap junctions), stromal arrangement, glandular/acinar structures	Spatial transcriptomics (Visium, CosMx, Xenium), multiplexed imaging (CODEX, MIBI, IMC), confocal microscopy	Assesses tissue-level normalization and microenvironmental context; identifies spatial niches; detects restoration of normal architecture	Good: Persistent architectural normalization suggests stable reversion; loss upon treatment withdrawal indicates plasticity	Specialized platforms required; costly; analytic complexity; requires tissue samples; limited to research settings currently
Circulating Indicators (Liquid Biopsy) [135,136]	cfDNA methylation patterns, exosomal cargo (RNA, proteins), circulating tumor cells (CTCs), circulating differentiation markers	Liquid biopsy platforms, cfDNA sequencing (methylation-specific), exosome isolation/analysis, CTC enumeration/characterization	Non-invasive; enables longitudinal monitoring without serial biopsies; early detection of molecular changes and relapses; serial sampling feasible	Moderate-Good: Serial cfDNA methylation can track epigenetic stability; Rising CTC counts or loss of differentiation markers in exosomes signals relapse	Sensitivity issues for low disease burden; indirect measure of tissue state; pre-analytic variables; expensive; interpretation complexity; not all cfDNA changes are functional
Clonal Tracking/Lineage Tracing [137]	Somatic mutations as clonal barcodes, lineage tracing constructs, mitochondrial DNA mutations	Deep sequencing of clonal markers, single-cell DNA sequencing, phylogenetic reconstruction	Tracks fate of individual cancer clones over time; determines if treated cells lose clonogenic potential or persist; distinguishes eradication vs. dormancy vs. reversion	Excellent: Can definitively determine if cancer clones are eliminated, enter dormancy, or undergo stable differentiation by tracking their fate over time	Requires sophisticated sequencing; complex analysis; primarily research tool; expensive; needs baseline tumor sampling for barcode identification
Proliferation/Dormancy Markers [127]	Ki-67 (proliferation), p21/p27 (cell cycle inhibitors), senescence markers (SA-β-gal, p16)	Immunohistochemistry, flow cytometry, senescence-associated β-galactosidase staining	Distinguishes quiescence/senescence from differentiation; helps rule out dormancy masquerading as reversion	Moderate: Sustained low Ki-67 with high differentiation markers suggests stable reversion; high Ki-67 return indicates reversibility	Requires serial measurement; cannot distinguish dormancy from true reversion alone; must be combined with differentiation/functional markers

Abbreviations: cfDNA = cell-free DNA; CTC = circulating tumor cell; FDG-PET = fluorodeoxyglucose positron emission tomography; H&E = hematoxylin and eosin; OXPHOS = oxidative phosphorylation; qPCR = quantitative polymerase chain reaction; SA-β-gal = senescence-associated beta-galactosidase; WGBS = whole-genome bisulfite sequencing; RRBS = reduced-representation bisulfite sequencing; 5-mC = 5-methylcytosine; 5-hmC = 5-hydroxymethylcytosine; CODEX = CO-Detection by indexing; MIBI = Multiplexed ion beam imaging; IMC = Imaging mass cytometry.

**Table 3:** Regulatory and Clinical Trial Considerations for Cancer Reversion Therapies.

Aspect	Conventional Cancer Therapy Trials	Cancer Reversion/Differentiation/Phenotypic Reversion Therapy Trials	Special Considerations for Reversion Approaches
Primary Endpoints [175,176,177]	Overall survival (OS), progression-free survival (PFS), objective response rate (ORR) as tumor shrinkage or delay of progression. (Established evidence)	Differentiation markers (e.g., lineage markers such as CD11b/CD14 in AML), phenotypic normalization (expression of normal tissue genes), functional recovery (hematopoietic improvement, restoration of differentiated function), molecular signatures, minimal residual disease (MRD) negativity, PLUS stability metrics: duration of response after treatment withdrawal (minimum 3–6 months post-cessation), epigenetic consolidation markers (DNA methylation changes), loss of tumor-initiating capacity. (Emerging/moderate evidence)	Need for novel endpoints capturing reversion not just growth inhibition; co-primary or surrogate endpoints require validation for clinical benefit; composite endpoints combining molecular + functional + stability measures; critical: distinguish transient plasticity from stable reversion by assessing post-treatment durability. (Speculative/moderate evidence)
Trial Design [178,179]	Randomized controlled trials (RCTs) with standard endpoints (OS, PFS, ORR). Short-term responses heavily weighted. (Established evidence)	Adaptive designs with biomarker-driven enrichment; early proof-of-concept studies with intermediate biomarker assessments; extended treatment cycles to allow phenotypic consolidation; longer mandatory follow-up periods (minimum 6–12 months post-treatment withdrawal for hematological malignancies, 12–24 months for solid tumors); possibly single-arm studies if reversion markers are validated; serial tissue/liquid biopsies to assess stability; withdrawal trials to test treatment-free remission. (Moderate evidence—some precedent in CML treatment-free remission trials)	Substantially longer follow-up needed to assess stability of reversion vs. reversible plasticity; design must allow measurement of durability, not just initial response; periodic assessment of phenotype over time (every 4–8 weeks initially, then every 3–6 months); incorporation of planned treatment withdrawal phase to test stability; enrichment for patients with biomarkers predicting stable vs. transient responses. (Speculative/moderate evidence)
Patient Selection [180,181]	Based on tumor histology, known genetic alterations, prior lines of therapy. (Established evidence)	Additional molecular/epigenetic profiling (mutation status, DNA methylation patterns, chromatin accessibility, expression of differentiation block regulators) to identify patients most likely to achieve stable reversion; assessment of baseline stemness signatures (Oct4, Sox2, Nanog); measurement of differentiation capacity (ex vivo differentiation assays); stratification by epigenetic landscape and mutational burden; potential exclusion of patients with secondary mutations preventing differentiation. (Emerging/moderate evidence)	Identification and validation of biomarkers predictive of stable vs. transient reversion; defining inclusion/exclusion criteria based on differentiation block mechanisms; possible stratification by epigenetic plasticity index; consideration of prior treatment history affecting differentiation capacity; development of companion diagnostics for patient selection. (Speculative)
Dosing Considerations [182,183]	Aim for maximum tolerated dose (MTD) where cytotoxic effect is required; standard continuous or intermittent dosing. (Established evidence)	Biologically effective dose (BED) may be well below MTD; focus on dose achieving phenotypic normalization rather than maximal cell killing; use of lower, sustained or pulsed dosing to promote differentiation vs. cytotoxicity; extended dosing duration (weeks to months) to allow epigenetic consolidation; maintenance dosing may be needed to reinforce differentiated state; tapering schedules to assess stability; pharmacodynamic endpoints (differentiation marker expression) guide dose optimization over pharmacokinetic parameters alone. (Moderate evidence)	Need to balance differentiation induction vs. toxicity; must consider epigenetic plasticity and potential for reversion, cessation may lead to relapse if consolidation incomplete; dosing schedule and duration may be more critical than dose intensity; prolonged low-dose exposure may be more effective than short high-dose for inducing stable epigenetic changes; patient-specific dose optimization based on molecular response. (Speculative/moderate evidence)
Combination Development [175,184]	Traditional phase I → II → III progression; combinations mainly to enhance cytotoxicity or overcome resistance. (Established evidence)	Reversion agents combined with other therapies (chemotherapy after differentiation, immunotherapy to eliminate partially differentiated cells, multiple epigenetic drugs for synergistic reprogramming); sequencing and scheduling critically important (e.g., differentiation pre-treatment followed by consolidation therapy); rational combinations targeting both differentiation induction AND stability maintenance; testing combinations that address: (1) induction of differentiation, (2) epigenetic consolidation, (3) elimination of undifferentiated resistant cells, (4) microenvironmental normalization. (Emerging/moderate evidence—some clinical precedent in AML with ATRA + chemotherapy)	Complex interactions: reversion agents may fundamentally alter cellular state affecting sensitivity to other treatments; risk of antagonism if differentiation causes cell cycle exit reducing chemotherapy sensitivity; risk of overlapping or novel toxicities; treatment schedule and sequence may dramatically impact outcomes; need for mechanism-based combination rationale not just empiric testing; incorporation of pharmacodynamic monitoring to optimize sequence/timing. (Speculative/moderate evidence)
Regulatory Pathways [177,178,185]	Well-established regulatory pathways; full approval typically requires OS/PFS benefit or strong evidence; accelerated/conditional approvals possible with validated surrogate endpoints. (Established evidence)	Reversion therapies may be eligible for accelerated or conditional approvals using novel surrogate endpoints (differentiation markers, MRD, functional recovery measures); possible breakthrough/RMAT designations facilitating use of non-traditional endpoints; requirement for post-marketing studies confirming clinical benefit; need to demonstrate not just response but stability/durability; potential for treatment-free remission as approvable endpoint (precedent: CML). (Moderate evidence—some precedent with ATRA in APL, imatinib in CML)	Need for early and continuous dialogue with regulators (FDA, EMA) to establish acceptable reversion endpoints and stability criteria; requirement for confirmatory studies demonstrating durability; regulatory agencies must accept differentiation/phenotypic markers as proxies for clinical benefit pending long-term survival data; development of guidance documents specific to reversion therapies; potential for adaptive licensing approaches allowing provisional approval with extended monitoring. (Speculative/moderate evidence)
Response Assessment [178,181]	RECIST/iRECIST for solid tumors; hematological response criteria (complete remission, partial remission); tumor burden measurement; imaging; minimal residual disease (MRD) in blood cancers. (Established evidence)	Addition of molecular markers of differentiation (lineage-specific transcription factors, surface markers, mature cell proteins); functional assays (restoration of normal cell phenotype and function, e.g., phagocytosis, metabolic normalization); advanced imaging of phenotype changes (metabolic PET, functional MRI); serial assessment of epigenomic status (DNA methylation, chromatin accessibility via liquid biopsy, cfDNA methylation); MRD with phenotypic characterization; ideally single-cell analyses and lineage tracing in research settings; stability assessment: serial measurements continuing 6–12 months post-treatment to confirm durability. (Emerging/moderate evidence)	Standardization urgently needed: consensus on which differentiation markers constitute meaningful reversion; ensure reproducibility across laboratories; validated, clinically feasible assays required; composite metrics combining molecular + morphological + functional components; development of novel imaging modalities for non-invasive phenotype monitoring; liquid biopsy approaches to avoid repeated tissue sampling; critical distinction: measures must differentiate stable reversion from transient plasticity, growth arrest, or senescence. (Speculative—significant work needed)
Long-term Monitoring [179]	Focus on recurrence, survival, late toxicities; standard follow-up periods (typically 2–5 years). (Established evidence)	Extended monitoring of stability of reverted phenotype: does differentiation persist after treatment cessation? Molecular relapse detection via serial liquid biopsies (cfDNA methylation patterns reverting to malignant signature); possibly regular tissue biopsies or bone marrow assessments in hematological malignancies; monitoring of immune status (for immune-mediated reversion approaches); epigenetic monitoring (methylation stability); metabolic monitoring (sustained metabolic normalization); potential need for indefinite surveillance given uncertainty about very late relapse; assessment of functional status and quality of life as indicators of stable normalization. (Moderate evidence—some data from CML treatment-free remission studies)	Novel surveillance protocols required: risk that after treatment cessation, malignant behavior may re-emerge months to years later; need to distinguish true stable reversion from prolonged dormancy; potential need for maintenance/consolidation therapy in subset of patients showing incomplete stability; longer follow-up periods essential in clinical trial design (minimum 2–5 years post-treatment cessation) to capture late relapse or loss of reversion; development of early warning biomarkers (e.g., rising stemness marker expression, loss of differentiation signatures) enabling intervention before overt relapse. (Speculative—largely unknown territory)
Toxicity Assessment [175,182]	Standard adverse event monitoring using CTCAE criteria; focus on cytotoxic organ toxicities (myelosuppression, mucositis, neuropathy); off-target effects. (Established evidence)	Special attention to differentiation syndrome (cytokine release, capillary leak—well-characterized in APL with ATRA); immune-related effects if combining with immunotherapy; altered metabolism-related toxicities (hypercalcemia with vitamin D derivatives); off-target epigenetic changes affecting normal tissues (potential for aberrant differentiation); lineage mis-specification risks; on-target/off-tumor effects (normal stem cells undergoing unwanted differentiation); long-term risks: potential for secondary malignancies from epigenetic therapies; monitoring for “paradoxical” toxicities (e.g., transient increases in circulating blasts during differentiation). (Moderate evidence)	Development of biomarkers for early differentiation syndrome detection and grading; monitoring of immune modulation and cytokine profiles; potential for unexpected toxicities related to cellular identity/lineage changes; assessment of long-term epigenetic risks in normal tissues (germline effects unlikely but somatic effects in proliferating normal tissues possible); specific concern: effects on normal stem cell compartments (hematopoietic, intestinal, etc.); need for extended safety follow-up given novel mechanism of action. (Speculative—largely unknown risks)
Cost Considerations [183,186]	Economic evaluation based on survival gains, cost of treatment, hospitalizations for complications, management of side effects, quality of life impacts. (Established evidence)	Potentially higher upfront development costs due to: biomarker assay development, extended trial durations, novel endpoint validation; higher per-patient monitoring costs (serial epigenetic profiling, specialized imaging, frequent assessments). However, potential for substantial long-term cost savings: outpatient-based therapy (lower toxicity reducing hospitalizations), improved quality of life (less intensive treatment), potential for treatment-free remission eliminating ongoing drug costs, reduced need for salvage therapies; value proposition: durable remissions with finite treatment duration vs. indefinite palliative therapy. (Moderate evidence—some data from CML treatment-free remission economic analyses)	Need for health economic modeling incorporating: value of quality-adjusted life years (QALYs) gained, reduced caregiver burden, productivity gains from less toxic therapy; payers may demand robust evidence of durable reversion and quality of life improvements before reimbursement; cost of extensive monitoring and long follow-up periods; potential for value-based pricing models (payment contingent on achieving stable reversion); reimbursement pathways may be challenged by non-standard endpoints; need for comparative effectiveness research vs. standard therapies; societal cost–benefit analysis considering potential for cure vs. chronic disease management. (Speculative—requires outcomes data)

Abbreviations: OS = Overall survival; PFS = Progression-free survival; ORR = Objective response rate; MRD = Minimal residual disease; AML = Acute myeloid leukemia; RCT = Randomized controlled trial; CML = Chronic myeloid leukemia; MTD = Maximum tolerated dose; BED = Biologically effective dose; ATRA = All-trans retinoic acid; APL = Acute promyelocytic leukemia; RMAT = Regenerative Medicine Advanced Therapy; FDA = Food and Drug Administration; EMA = European Medicines Agency; RECIST = Response Evaluation Criteria in Solid Tumors; iRECIST = Immune RECIST; PET = Positron emission tomography; MRI = Magnetic resonance imaging; cfDNA = Cell-free DNA; CTCAE = Common Terminology Criteria for Adverse Events; QALY = Quality-adjusted life year.

**Table 4:** (A) Evidence-Based Novel Combination Approaches for Enhancing Cancer Reversion; (B) Proposed Novel Combinations Requiring Experimental Validation.

(**A**)						
**Combination Strategy**	**Mechanistic Rationale**	**Cancer Types**	**Development Status**	**Potential Advantages**	**Stability Enhancement Mechanism**	**Challenges**
Epigenetic Modifiers + Immunotherapy [187,188]	DNMTi/HDACi upregulate tumor antigens, MHC-I, cancer-testis antigens, enhancing immune detection; immune pressure may drive/maintain differentiation	Melanoma, NSCLC, solid/liquid tumors	Phase I/II trials	Converts “cold” to “hot” tumors; boosts checkpoint blockade responses; immune surveillance may enforce phenotypic stability	Immune selection pressure eliminates dedifferentiated clones; sustained immune memory prevents relapse	Timing/sequencing critical; irAEs; PD-L1 induction may enhance or impair efficacy
HDAC Inhibitors + DNMT Inhibitors [188,189,190]	Synergistic epigenetic remodeling: DNA hypomethylation + histone acetylation reactivates silenced tumor suppressors	AML, myelodysplastic syndromes, colon cancer, lymphomas	Phase II/III trials	Deep epigenetic reprogramming; DNA methylation changes provide stability; histone acetylation provides initial opening	Dual epigenetic hits may achieve more stable chromatin remodeling than either alone	Myelosuppression; limited solid tumor activity; overlapping toxicities
Differentiation Agents + Sequential Chemotherapy [191]	Differentiation restores chemosensitivity by altering cell cycle, chromatin state, DNA repair capacity	AML, leukemia	Preclinical/translational	May improve remission durability by eradicating residual immature cells; chemotherapy eliminates cells unable to complete differentiation	Differentiation followed by selective elimination of incompletely differentiated cells	Sequencing critical; risk of antagonism if differentiation causes cell cycle exit reducing chemo sensitivity; timing window narrow
Epigenetic Modifiers + Antibody-Drug Conjugates [192]	Epigenetic priming enhances ADC target antigen expression (e.g., ICAM1), uptake, anti-tumor effect	Melanoma PDX, lung cancer	Preclinical (PDX studies)	Potentiates ADC response in low-antigen tumors; combined differentiation + targeted cytotoxicity	Epigenetic priming may produce partial differentiation; ADC eliminates resistant undifferentiated cells	Toxicity from dual agents; off-target antigen induction; requires validation; dosing/timing optimization
(**B**)						
**Combination Strategy**	**Proposed Rationale**	**Cancer Types**	**Current Status**	**Hypothetical Advantages**	**Potential Stability Mechanisms**	**Unknown Risks/Challenges**
Differentiation Agents + Senolytic Drugs	Differentiation therapy may induce senescence in subset of cells; senolytics eliminate senescent cells to prevent SASP-mediated tumor promotion and potential dedifferentiation	Pancreatic, prostate, solid tumors	Conceptual; no studies	Could eliminate dormant/resistant senescent cells that may harbor dedifferentiation capacity; reduce SASP	Removal of senescent cells may eliminate reservoir for phenotypic reversion; SASP factors can induce stemness	No validated senescence biomarkers in solid tumors; senolytics may harm beneficial senescent non-malignant cells; timing critical
Microenvironmental Modulators + Metabolic Reprogramming	Normalize tumor stroma/vasculature while redirecting tumor metabolism (e.g., forcing oxidative metabolism in glycolytic tumors)	Breast, ovarian, lung	Theoretical; limited studies	Targets both extrinsic (microenvironment) and intrinsic (metabolism) resistance pathways; metabolic reprogramming may lock in differentiated state	Differentiated cells require oxidative metabolism; forcing metabolic shift may prevent dedifferentiation to glycolytic stem-like state	High complexity; drug delivery challenges; timing/sequencing unknown; potential for metabolic plasticity allowing adaptation
Stromal Reprogramming + Anti-angiogenic Therapy	Remodel cancer-associated fibroblasts from pro-tumor to anti-tumor phenotype + normalize vasculature to improve drug delivery and reduce hypoxia-induced stemness	Pancreatic, colorectal, breast	Early concept	Could reduce immune exclusion, increase reversion agent penetration; normalized stroma may enforce differentiated phenotype	Fibroblasts produce differentiation factors; normal vasculature reduces hypoxia-driven dedifferentiation	Narrow therapeutic window; fibroblast reprogramming methods limited; anti-angiogenics often cause hypoxia; potential tumor adaptation
RNA-Based Therapeutics + Nanoparticle Delivery	Use miRNAs/siRNAs to reprogram transcriptional networks involved in differentiation; nanoparticles enable targeted delivery	Hepatocellular carcinoma, glioblastoma	Preclinical (early nanoparticle studies, not in reversion context)	Targeted reprogramming of master regulator pathways; restoration of miRNA networks characteristic of differentiated cells	miRNAs like let-7, miR-200 family enforce differentiated state by suppressing stemness factors; stable expression may prevent dedifferentiation	Delivery barriers to solid tumors and CNS; off-target gene silencing; immune responses to RNA; transient effects unless stably integrated
Engineered Cell Therapies + Differentiation Inducers	CAR-T/NK cells clear bulk tumor burden + differentiation inducers reprogram residual cells; CAR-T cells may deliver differentiation factors locally	Leukemias, neuroblastoma, potentially solid tumors	Conceptual; CAR-T and differentiation agents used separately	Addresses tumor heterogeneity: eliminates rapidly proliferating cells while reprogramming resistant subpopulations; immune elimination of dedifferentiated clones	Continuous immune surveillance by long-lived CAR-T cells may prevent outgrowth of dedifferentiated clones; CAR-T cells engineered to secrete differentiation factors provide sustained local delivery	Complex manufacturing; unpredictable interactions between cell therapy and differentiation agents; CAR-T exhaustion/persistence issues; cytokine release syndrome; solid tumor penetration limited

Abbreviations: DNMTi = DNA methyltransferase inhibitor; HDACi = Histone deacetylase inhibitor; NSCLC = Non-small cell lung cancer; irAE = Immune-related adverse event; MHC-I = Major histocompatibility complex class I; ADC = Antibody-drug conjugate; PDX = Patient-derived xenograft; ICAM1 = Intercellular adhesion molecule 1; SASP = Senescence-associated secretory phenotype; CAR-T = Chimeric antigen receptor T-cell; CAR-NK = Chimeric antigen receptor natural killer cell.

## Data Availability

No new data were created or analyzed in this study. Data sharing is not applicable to this article.

## Abbreviations

3D	Three-dimensional
AML	Acute myeloid leukemia
APL	Acute promyelocytic leukemia
ATRA	All-trans retinoic acid
BCC	Basal cell carcinoma
BET	Bromodomain and extra-terminal
CAR-T	Chimeric antigen receptor T-cell
CML	Chronic myeloid leukemia
CRISPR	Clustered regularly interspaced short palindromic repeats
CSC	Cancer stem cell
CTC	Circulating tumor cell
DNMT	DNA methyltransferase
ECM	Extracellular matrix
EMT	Epithelial–mesenchymal transition
FDA	Food and Drug Administration
HDAC	Histone deacetylase
HIF	Hypoxia-inducible factor
iPSC	Induced pluripotent stem cell
MErT	Mesenchymal to epithelial reverting transition
NSCLC	Non-small cell lung cancer
OS	Overall survival
PDX	Patient-derived xenograft
PFS	Progression-free survival
PPARγ	Peroxisome proliferator-activated receptor gamma
PROTAC	Proteolysis-targeting chimera
scRNA-seq	Single-cell RNA sequencing
TAM	Tumor-associated macrophage
TNBC	Triple-negative breast cancer
VEGF	Vascular endothelial growth factor
VDR	Vitamin D receptor
